# Anoctamin 2-chloride channels reduce simple spike activity and mediate inhibition at elevated calcium concentration in cerebellar Purkinje cells

**DOI:** 10.1371/journal.pone.0247801

**Published:** 2021-03-02

**Authors:** Friederike Auer, Eliana Franco Taveras, Uli Klein, Céline Kesenheimer, Dana Fleischhauer, Frank Möhrlen, Stephan Frings

**Affiliations:** Department of Animal Physiology, Centre for Organismal Studies, University of Heidelberg, Heidelberg, Germany; Doheny Eye Institute/UCLA, UNITED STATES

## Abstract

Modulation of neuronal excitability is a prominent way of shaping the activity of neuronal networks. Recent studies highlight the role of calcium-activated chloride currents in this context, as they can both increase or decrease excitability. The calcium-activated chloride channel Anoctamin 2 (ANO2 alias TMEM16B) has been described in several regions of the mouse brain, including the olivo-cerebellar system. In inferior olivary neurons, ANO2 was proposed to increase excitability by facilitating the generation of high-threshold calcium spikes. An expression of ANO2 in cerebellar Purkinje cells was suggested, but its role in these neurons remains unclear. In the present study, we confirmed the expression of *Ano2* mRNA in Purkinje cells and performed electrophysiological recordings to examine the influence of ANO2-chloride channels on the excitability of Purkinje cells by comparing wildtype mice to mice lacking ANO2. Recordings were performed in acute cerebellar slices of adult mice, which provided the possibility to study the role of ANO2 within the cerebellar cortex. Purkinje cells were uncoupled from climbing fiber input to assess specifically the effect of ANO2 channels on Purkinje cell activity. We identified an attenuating effect of ANO2-mediated chloride currents on the instantaneous simple spike activity both during strong current injections and during current injections close to the simple spike threshold. Moreover, we report a reduction of inhibitory currents from GABAergic interneurons upon depolarization, lasting for several seconds. Together with the role of ANO2-chloride channels in inferior olivary neurons, our data extend the evidence for a role of chloride-dependent modulation in the olivo-cerebellar system that might be important for proper cerebellum-dependent motor coordination and learning.

## Introduction

The activity of a neuronal network depends on the excitability of the neurons within this network and on the modulation of this excitability. Excitability of individual neurons can either be increased or decreased, and both changes will affect the output signal of the network. Calcium-activated chloride channels are of special interest in this context, because they can either enhance or attenuate neuronal excitability in various ways (see [1] for recent review). The intracellular chloride concentration differs between neurons and can vary between different compartments of the same neuron (reviewed in [2]). Consequently, calcium-induced chloride currents can be either hyperpolarizing or depolarizing. This characteristic feature of chloride currents leads to various modes of modulation through which excitability can be affected.

The calcium-activated chloride channel Anoctamin 2 (ANO2) was found to decrease excitability in neurons of the hippocampus [3], thalamus [4], lateral septum [5], and central lateral amygdala [6], whereas excitability is increased in olfactory receptor neurons [7,8] and the olivo-cerebellar system [9,10]. The enhancing effects of ANO2 in the olivo-cerebellar system has been associated with impairments in motor coordination and learning tasks observed in ANO2-deficient mice (*Ano2*^*-/-*^) [9,11]. However, while the enhancement of climbing fiber signals by ANO2 is well established, the effect of ANO2 on Purkinje cell excitability remains controversial.

In climbing fibers, ANO2 facilitates rapid repolarization after high-threshold spikes and, therefore, enables faster spiking [9]. Additionally, ANO2 has been suggested to attenuate GABAergic inhibition from molecular layer interneurons onto Purkinje cells [10]. Because climbing fibers are also involved in this process [12], it is still unclear whether ANO2 in Purkinje cells or in climbing fibers is responsible for the reduction of GABAergic signals. In the present study, we specifically examine the contribution of ANO2 to the modulation of excitability in Purkinje cells. To this end, we analyze the excitability of Purkinje cells in acute tissue slices, uncoupled from climbing fiber input, and compare wildtype to *Ano2*^*-/-*^ mice. Although we used a global *Ano2*^*-/-*^ mouse for this study, our experimental design revealed local effects of ANO2 on Purkinje cell neurons and illustrated the impact of ANO2 solely on the network of the cerebellar cortex.

Our results confirm, that ANO2 is expressed in Purkinje cells. We discovered that ANO2 decreases instantaneous simple spike activity of Purkinje cells following strong current injections and current injections close to the activation threshold of Purkinje cells. Moreover, we report an ANO2-mediated attenuation of GABAergic inhibition for a few seconds after depolarization of Purkinje cells. Combined with the proposed role of ANO2 in climbing fibers [9], we integrate these new findings into the concept of chloride-dependent modulation of network activity in the cerebellar cortex.

## Materials and methods

### Animals

Eight to twelve weeks old male C57BL/6N mice (Charles River Laboratories, Wilmington, Massachusetts, USA) and *Ano2*^*-/-*^ mice [8] (kindly provided by Dr. Thomas Jentsch, FMP, Berlin, Germany) were used for all the experiments. Mice were kept in the Interfaculty Biomedical Facility (IBF) of the Heidelberg University (Germany) under SPF conditions. Mice were anesthetized by isoflurane inhalation (Baxter, Deerfield, Illinois, USA) and then put to death with an overdose isoflurane. Animal housing and all experimental procedures were carried out in compliance with the guidelines for the welfare of experimental animals as stipulated by the Federal Republic of Germany. Animal experiments were approved by the Regierungspraesidium Karlsruhe, Germany (approval numbers T-04/18, T-05/18).

### Quantitative real-time PCR (qPCR)

Immediately after sacrificing the animals, tissue samples were collected and flash-frozen in liquid nitrogen. Total RNA was extracted using either the MagJET RNA Kit (Thermo Fisher Scientific, Waltham, Massachusetts, USA) or the RNeasy Mini Kit (Qiagen, Hilden, Germany) according to the manufacturer’s instructions. cDNA was synthetized with 50 ng of each RNA preparation using the SuperScript™ VILO™ Master Mix (Thermo Fisher Scientific) according to the manufacturer’s instructions. Quantitative real-time PCR (qPCR) was performed with StepOne Real Time PCR System (Applied Biosystems, Foster City, California, USA) using the Fast Start Universal SYBR Green Master (ROX) (Roche, Basel, Switzerland). In every run, a negative control and duplicates for each primer pair were used. A melting-curve analysis was included in every qPCR run. YWHAZ and Gusb were chosen as housekeeping genes, as they are supposed to be most consistently expressed across different regions of the mouse brain [13].

The CT-values, obtained from the StepOne Real Time PCR System, were analyzed using the relative quantification method [14] which is implemented in the REST 2009 software (Qiagen). Data obtained from inferior olivary tissue samples were chosen as reference sample. Primer efficiencies were calculated using the Miner-Algorithm [15]. Since the resulting fold-expression values are not normally distributed, the 68% confidence was calculated in REST 2009 using a randomization approach and was used as a measure of variation of the samples.

Primer pairs used were (5’-> 3’): ano2/f TTTATGATTGCCCTGACGTTCTC, ano2/r GAGGTTGATGATGACTGCTGTT, ywhaz/f TAGGTCATCGTGGAGGGTCG, ywhaz/r GAAGCATTGGGGATCAAGAACTT, gusb/f CCGACCTCTCGAACAACCG, gusb/r GCTTCCCGTTCATACCACACC.

### *In situ* hybridization (ISH)

cDNA was synthesized from extracted RNA from olfactory epithelium or cerebellum using the SuperScript™ VILO™ Master Mix (Thermo Fisher Scientific). cDNA fragments were amplified through PCR using two sets of primers (ano2_1: primer pair used for qPCR (see above), ano2_2/f ACAACGGGACACTACATGGC, ano2_2/r TCGCTGATGTCAGTGGGGAT). The PCR products were purified using the PCR Cleanup Kit (Macherey-Nagel, Düren, Germany) and ligated in a pGEM-T Vector (Promega, Madison, Wisconsin, USA). Once ligated, plasmids were transformed through heat-shock into JM109 High-Efficiency E. Coli Competent Cells (Promega). Positive recombinants were incubated in LB-Medium and the samples were sequenced in Microsynth SeqLab (Göttingen, Germany) after minipreparation. After confirming the proper ligation of the insert and match with *Ano2* mRNA through BLAST, the plasmids were linearized using the enzymes NcoI or NdeI (Thermo Scientific). The linearized vectors were finally incubated for 2 h at 37°C with the RNA-Polymerases SP6 or T7 using the DIG-RNA-Labeling Mix (Roche) to synthesize the sense (control) and the antisense hybridization probes, respectively.

C57BL/6N mice were transcardially perfused with 4% paraformaldehyde (PFA). After perfusion, heads were fixed for 2 h in 4% PFA and then washed with PBS (150 mM NaCl, 8.1 mM Na_2_HPO_4_ x 2H_2_0, 1.9 mM NaH_2_PO_4_ x H_2_0, pH adjusted to 7.4) for 5 min and submerged in 0.5 M EDTA for at least a week at 4°C for decalcification, until cranial bones softened. To dewater the tissue, heads were put in 10% sucrose solution (2–3 h, RT), 30% sucrose solution (overnight, 4°C) and in a 1:1 mixture of Tissue-Tek® O.C.T.™ Compound (2–3 h, RT) (Sakura Finetek, Tokyo, Japan) and 30% sucrose solution (2–3 h, RT), before being embedded in Tissue-Tek® O.C.T.™ Compound only. Sagittal and coronal sections (16 μm) were sliced with a cryostat (CM3050, Leica, Wetzlar, Germany), set at a chamber temperature of -22°C and an object temperature of -20°C. The sections were collected on SuperFrost Plus adhesion slides (Thermo Fisher Scientific) and were left to dry for no longer than 30 min and stored at -20°C.

As a pretreatment of the tissue, slices were fixed in 4% PFA for 10 min and then washed 2x 5 min in PBS. Afterwards, slices were digested with 50 μg/ml Proteinase K in PK-Buffer (50 mM TRIS/HCl and 5 mM EDTA adjusted to pH 8.3) for 5 min at 37°C and washed twice in PBS for 5 min. The samples were acetylated with 0.1 M triethanolamine solution containing 0.25% acetic acid (pH 8) for 10 min. After two washing steps with PBS, slices were incubated for 4–6 h in a prehybridization buffer (25% 5x SSC, 10% 1x Denhardt’s solution, 50% deionized formamide, 0.1% Tween-20, 500 μg/ml Herring Sperm DNA; for 20xSSC: 3 M NaCl, 0.3 M trisodium citrate, pH 7.0) before being incubated overnight with the hybridization buffer (25% 5x SSC, 10% 1x Denhardt’s solution, 50% deionized formamide, 0.1% Tween-20) containing 350–450 ng of the hybridization probe at 55°C. On the next day, the slices were washed with 5x SSC at 55°C, 0.2x SSC at 55°C and 0.2x SSC at RT for 30 min each. Slices were washed 5 min in PBS with 0.1% Triton X-100 and 0.5% BSA (PBT) and then blocked with a blocking solution containing 5% BSA in PBT for 1 h at RT. After blocking, the anti-Digoxigenin-AP antibodies (Roche, dilution 1:5000 in blocking solution) were added and incubated for 2 h at RT. Slices were washed 2x 30 min in PBT and incubated in NBT/BCIP coloration solution (Ready-to-use tablets, Roche) until a staining was visible for the antisense but not the sense hybridization probe (30 min to 4 h or overnight). Slices were washed with PBS to stop the reaction. The inferior olivary nucleus (coronal slices) or the inferior olivary nucleus and the hippocampus (sagittal slices) served as positive controls in each staining. Every staining procedure included stainings with the sense hybridization probe as negative control.

For the combined ISH/immunohistochemistry (IHC) staining (modified from [16]) samples were blocked in CT solution (20% ChemiBlocker (Merck, Darmstadt, Germany), 0.5% Triton X-100 in 1x PBS pH 7.4) for 2 h at RT after the SSC washing steps. The samples were then incubated overnight with anti-digoxigenin antibody (Roche, dilution 1:5000) and the primary antibody Calbindin D-28K (Swant Cat# CB-38a, RRID: AB_10000340, Lot# 9.03, Marly, Switzerland, polyclonal, raised in rabbit, dilution 1:1000; [17]) in CTA solution (20% Chemiblocker, 0.5% Triton X-100, 0.05%, NaN_3_ in 1x PBS pH 7.4). On the next day, the slices were washed 3x 10 min with PBT and incubated with the secondary antibody goat anti-rabbit conjugated with Alexa Fluor 568 (Thermo Fisher Scientific Cat# A-11011, RRID:AB_143157, Lot# 1558746, dilution 1:1000) for at least 2 h in C solution (20% ChemiBlocker in 1x PBS pH 7.4). Additionally, the anti-Digoxigenin antibody was added to prevent losing signal. Samples were washed twice in PBT for 30 min and incubated in NBT/NCIP coloration solution. Slices were washed with PBS, 0.3 μM DAPI (Sigma-Aldrich Cat# 32670, St. Louis, Missouri, USA) was added to the samples for 10 min and washed again with PBS. Lastly, a coverslip was mounted with Aqua Poly/Mount (Polysciences, Warrington, Pennsylvania, USA). For each staining, a negative control using only the secondary antibody was performed.

Images of ISH stainings were taken with an Eclipse 90i microscope (Nikon, Minato, Japan) using the brightfield camera DS-Ri1 (Nikon) and IHC stainings were captured with the confocal laser scanning system D-Eclipse C1 (Nikon). Images were captured with the imaging software NIS-Elements AR 4.00.12 (RRID:SCR_014329, Nikon). The following objectives were used: 4x (Nikon Plan Fluor 4x/NA 0.13) and 60x (Nikon Plan Apo VC 60x/NA 1.40 Oil). Contrast and brightness of the images were adjusted with CorelPHOTO-PAINT 2017 (Corel Corporation, Ottawa, Canada).

### Free-floating immunohistochemistry

The cerebellum was removed and fixed for 30 min in 4% paraformaldehyde (PFA) solution. The cerebellum was cut in ice-cold PBS into 200 μm-thick slices with a vibratome (VT1000S, Leica). The slices were fixed again for 30 min in 4% PFA solution. After washing for 4x 10 min with PBS, slices were incubated for 2 h in a blocking solution containing 5% goat serum (Sigma-Aldrich), 0.5% Triton X-100 and 0.05% NaN_3_. Primary antibodies were applied overnight (diluted in blocking solution) and sections were washed again 4x 10 min in PBS with 0.5% Triton X-100 (PBST). Unless the primary antibody was directly coupled with a fluorescent tag, the slices were incubated for 2 h with the secondary antibodies (diluted in blocking solution), which were conjugated to a fluorescent tag. After washing 4x 10 min with PBST, slices were mounted on glass slides with Aqua-Poly/Mount (Polysciences). The slices were kept on a shaker at RT in the dark for the entire procedure.

Cerebellar Purkinje cells were visualized with an antibody against Calbindin D-28K [18] (Swant, dilution 1:1000) and molecular layer interneurons together with Purkinje cells were stained with Parvalbumin [18,19] (Synaptic Systems Cat# 195 006, RRID:AB_2619887, Göttingen, Germany, polyclonal, raised in chicken, dilution 1:250; [20]). GABAergic synapses were targeted with an antibody specific for the Vesicular GABA transporter [21] (VGAT, Synaptic Systems Cat# 131 005, RRID:AB_1106810, polyclonal, raised in guinea pig, dilution 1:500; [22]). To visualize climbing fiber synapses, antibodies against the Vesicular glutamate transporter 2 (VGluT2, Millipore Cat# MAB5504A4, RRID:AB_11210701, Lot# 2914310, Burlington, Massachusetts, USA, monoclonal, raised in mouse, dilution 1:250; [23]), which is, in the molecular layer, specifically expressed in synapses of climbing fibers [24]. This antibody was directly coupled to Alexa Fluor 488, so no secondary antibody was needed for the detection of fluorescent signals. The secondary antibodies used were goat anti-rabbit conjugated with Alexa Fluor 405 (Thermo Fisher Scientific Cat# A-31556, RRID:AB_221605, dilution 1:500), goat anti-rabbit conjugated with Alexa Fluor 488 (Molecular Probes, Eugene, Oregon, USA, Cat# A-11008, RRID:AB_143165, Lot# 1515529, dilution 1:2000), goat anti-rabbit conjugated with Alexa Fluor 568 (Molecular Probes, Cat# A-11011, RRID:AB_143157, Lot# 1558746, dilution 1:2000), goat anti-chicken conjugated with Alexa Fluor 568 (Abcam, Cambridge, UK, Cat# ab175711, RRID:AB_2827757, dilution 1:1000) and goat anti-guinea pig conjugated with Alexa Fluor 568 (Molecular Probes Cat# A-11075, RRID:AB_141954, dilution 1:1000). Unless the secondary antibody with Alexa Fluor 405 was used, 0.3 μM DAPI was added to the secondary antibody dilution to visualize cell nuclei. A negative control using only the secondary antibody was performed for each staining, except for the tagged VGluT2 antibody.

Images of IHC stainings were taken with an Eclipse 90i microscope (Nikon) using the confocal laser scanning system D-Eclipse C1 (Nikon). Images were captured with the imaging software NIS-Elements AR 4.00.12 (Nikon) using a 40x (Nikon Plan Fluor 40x/NA 1.30 Oil) and a 60x (Nikon Plan Apo VC 60x/NA 1.40 Oil) objective. For analysis of Purkinje cell morphology, density of Purkinje cells and molecular layer interneurons, z-stacks (range 4–9 μm) were taken with a 40x objective and the maximum intensity projections were analyzed with ImageJ (RRID:SCR_003070, NIH, Bethesda, Maryland, USA). Interneurons were identified by subtracting the calbindin signal from the parvalbumin signal [25]. Because the cell bodies of Purkinje cells are aligned in a row, density of Purkinje cells was defined as cell bodies per mm. On the other hand, density of molecular layer interneurons was defined as cells per volume of the molecular layer, since they are scattered throughout the molecular layer. For counting climbing fiber and GABAergic synapses, images were pre-processed with iLastik (RRID:SCR_015246, HCI, Heidelberg, Germany) and then further analyzed with ImageJ. Images were taken at 60x magnification and a zoom factor ranging from 1.0 to 2.0. As climbing fiber synapses are located primarily on the main dendrites, only synapses in the proximal half of the molecular layer were counted. To analyze the distribution of climbing fiber synapses, distances between adjacent synapses on or next to the same dendrite were measured. Data are presented as curves describing the shape of the underlying histogram (bin size 0.5 μm). Contrast and brightness of the images shown in this study were adjusted with CorelPHOTO-PAINT 2017 (Corel Corporation).

### Electrophysiology

The cerebellum was removed immediately after sacrificing the animal and was mounted on the stage of a vibratome (VT1000S, Leica) with cyanoacrylate. The tissue was covered with artificial cerebrospinal fluid (ACSF) containing 134.6 mM NaCl, 21 mM NaHCO_3_, 3.4 mM KCl, 0.6 mM NaH_2_PO_4_, 1 mM MgCl_2_, 2.5 mM CaCl_2_. The pH was adjusted to 7.4 with HCl and osmolarity was set between 305 and 320 mOsmol/kg. During slicing, the temperature of the ACSF was set to 32–35°C. Acute parasagittal cerebellar slices (200–250 μm) were obtained and incubated in a holding chamber at 35°C for at least 30 min, as previously reported [26,27]. During the experiments, the holding chamber was kept at RT (25–28°C). All solutions were constantly oxygenated with 95% O_2_/5% CO_2_.

For electrophysiological experiments, the slices were transferred onto the stage of an upright microscope (Nikon Eclipse E600FN). The recording chamber was continuously perfused with oxygenated ACSF at a speed of 1–2 ml/min at RT. Purkinje cells were identified by their morphology using a brightfield camera (DS-Ri1, Nikon) and a water-immersion objective (Nikon Fluor 40x/0.80W). For all experiments, borosilicate glass micropipettes (Science Products, Hofheim, Germany, GB-150F 10) were produced with a horizontal puller (P97; Sutter Instruments, Novato, California, USA) and polished with a microforge to a resistance of 3–5 MΩ. Electrical signals were recorded using a patch-clamp amplifier (EPC 8, HEKA Electronics, Lambrecht, Germany) and the WinWCP Software (RRID:SCR_014713, Version 5.5.2, University of Strathclyde, Glasgow, UK).

For current-clamp experiments, micropipettes were filled with a K-methane sulfonate based internal solution containing 150 mM K-methane sulfonate, 5 mM KCl, 0.1 mM Cs-EGTA, 10 mM Na-HEPES, 3 mM MgATP, 0.4 mM NaGTP, pH 7.4 with KOH, osmolarity 302 mOsmol/kg. This resulted in a reversal potential for chloride (E_Cl_) of -87 mV that has previously been reported to reflect the physiological conditions of Purkinje cells [28]. Mature Purkinje cells have been shown to express KCC2 but not its counterpart NKCC1 [29,30], leading to a pronounced chloride extrusion and, therefore, a low E_Cl_.

After obtaining the whole-cell configuration, the cell was held at -70 mV for 5 min. Two test pulses (-5 mV and -10 mV, 0.5 s) were induced in voltage-clamp mode to analyze series and input resistance off-line. Spontaneous activity was recorded in current-clamp mode (0 pA, 1 min). To examine the intrinsic excitability of Purkinje cells, the cells were held near -70 mV with hyperpolarizing current injections that were on average comparable between wildtype and *Ano2*^*-/-*^ mice (wt: -496.0 ± 123.5 pA; *Ano2*^*-/-*^: -506.4 ± 105.8 pA). Two hyperpolarizing (-200 pA and -100 pA) and five depolarizing current pulses (200–1000 pA) were applied for 5 s to monitor the excitability of Purkinje cells in dependence of the amplitude of the current injection. For analysis, three different stages of activation were defined: threshold activation (current injection of 200 pA), moderate activation (600 pA) and strong activation (1000 pA). Each current injection was followed by a test pulse (-50 pA, 1 s) to monitor input resistance during the recording. Current pulses were separated by a break of 2 min. Data were filtered at 3 kHz and sampled at 20 kHz. Series resistance was 5–10 MΩ and was not compensated. To examine the role of chloride currents for excitability of Purkinje cells, measurements were repeated with a slightly modified intracellular solution, where the chloride concentration was adjusted to 23 mM chloride (132 mM K-methane sulfonate, 23 mM KCl, 0.1 mM Cs-EGTA, 10 mM Na-HEPES, 3 mM MgATP, 0.4 mM NaGTP, pH 7.4 with KOH, osmolarity 304 mOsmol/kg) to raise E_Cl_ to -48 mV which corresponds to the mean simple spike threshold (S2D Fig) that was recorded in the previous experiments. Unless otherwise stated, gabazine (20 μM, Tocris Bioscience) was applied by bath application in all experiments.

For voltage-clamp experiments, micropipettes were filled with an internal solution where K-methane sulfonate was exchanged by Cs-methane sulfonate (150 mM Cs-methane sulfonate, 5 mM KCl, 0.1 mM Cs-EGTA, 10 mM Na-HEPES, 3 mM MgATP, 0.4 mM NaGTP, pH 7.4 with CsOH, osmolarity 295 mOsmol/kg). Since hydrogencarbonate permeability has been reported for Anoctamin-chloride channels [31,32], 0.5 mM of the pH-sensitive dye HPTS (Sigma-Aldrich Cat# H1529; [33]) was added to the intracellular solution in all experiments to visualize Purkinje cell dendrites. Additionally, HPTS fluorescence was used in a preliminary experiment to confirm that ANO2 conducts mainly chloride instead of hydrogencarbonate [31,32] in our experiments. After obtaining the whole-cell configuration, cells were held at -70 mV for 10 min to ensure uniform distribution of HPTS in the dendritic tree. Inhibitory postsynaptic currents (IPSCs) were induced using a slightly modified version of the protocol from [12]. A stimulation pipette (2–2.5 MΩ) filled with ACSF was placed in the molecular layer. The HPTS-fluorescence signal was used to detect non-fluorescent areas between the dendrites of the Purkinje cell of interest, which were used to predict the positions of molecular layer interneurons. Electrical stimulation using a constant current source (DS7A; Digitimer, Welwyn Garden, UK) induced evoked IPSCs (eIPSCs) every 15 s (800 μA, 100 μs, interstimulus interval 50 ms) at a holding potential of -60.5 mV. Experiments on the reduction of inhibition were performed, as previously described [12], with a train of five depolarizing pulses via the patch pipette (-70.5 mV to -20.5 mV for 1 s at 0.5 Hz). A test pulse (5 mV, 0.5 ms) was induced before each stimulation to monitor the series resistance, which was 5–9 MΩ in all experiments. Series resistance was not compensated. Data were filtered and sampled at 3 kHz. ACSF included the AMPA/Kainate receptor antagonist CNQX (10 μM, Tocris Bioscience, Bristol, UK), the NMDA antagonist D-AP5 (40 μM, Tocris Bioscience), the GABA_B_ antagonist CGP 55845 (2 μM, Tocris Bioscience) and the CB_1_ antagonist AM 251 (2 μM, Tocris Bioscience) in all recordings. For control experiments, tetrodotoxin (TTX; 0.5 μM, Abcam) or gabazine (20 μM, Tocris Bioscience) were applied by bath application.

Electrophysiological data were analyzed off-line using Origin (RRID:SCR_014212, Version 9.0, OriginLab, Northampton, Massachusetts, USA) and Spike2 (CED, RRID:SCR_000903, Cambridge, UK). In all experiments, measurements were discarded if the series resistance changed by more than 20% during the recording. Current clamp measurements of spontaneous simple spike activity were only included in analysis, if continuous simple spike firing could be observed for 1 min. The first eight seconds were used for calculation of simple spike frequency in all recordings. Current clamp measurements with current injections were only used for analysis if the input resistance varied by less than 25% during recording and the holding potential stayed between -65 and -75 mV. Simple spikes were counted in the first 500 ms of each current injection step. For the analysis of spike frequency adaptation, interspike intervals (ISIs) of the first sixteen simple spikes after current injections were examined. The amplitude of the hyperpolarization after the current injection was calculated as the difference between the minimum membrane potential during hyperpolarization and the resting membrane potential before the current injection.

The eIPSC amplitudes were calculated as difference between the maximal current during the eIPSC and the holding current immediately before the stimulation. To compare eIPSC measurements of different cells, eIPSC amplitudes were normalized to the average eIPSC amplitude before the depolarization pulse (baseline). Time constant τ for recovery of eIPSC amplitude after depolarization was obtained by using the following model for exponential regression:

$$f(t)=y_{0}+a(1-e^{-\frac{t}{\tau }}).$$ (1)

### Statistics

Statistics were performed using Origin and SigmaPlot (RRID:SCR_003210, Version 13, Systat Software, San José, California, USA). All data sets were tested for normal distribution (Shapiro-Wilk test) prior to statistical analysis. Not normally distributed data were analyzed with the nonparametric Wilcoxon-Mann-Whitney test or the Kruskal-Wallis test. Normally distributed data were analyzed using Student’s unpaired two-tailed *t*-test with Welch correction or one-way ANOVA with Holm-Sidak’s multiple-comparison test, unless otherwise stated. Two-way ANOVA with Holm-Sidak’s multiple-comparison test was used for the comparison of interspike intervals. Significance level was set to 0.05. Sample sizes were defined as n = number of images or cells from N animals. Data are presented as box plots, with the line indicating the median, the square indicating the mean and the whiskers representing 1.5x interquartile range (IQR). In the figure legends, normally distributed data are presented as mean ± SD otherwise as median with IQR.

## Results

### *Ano2* is expressed in cerebellar Purkinje cells

An expression of ANO2 in Purkinje cells has previously been reported using immunohistochemistry [10,11]. On the other hand, no fluorescence was detected in Purkinje cells of another global ANO2-knockout mouse model that expresses farnesylated mCherry instead of ANO2 [9]. This raised a controversy about whether ANO2 is indeed expressed in Purkinje cells and, to examine this question, we performed qPCR and *in situ* hybridization experiments to investigate the expression of *Ano2*-mRNA in cerebellar tissue. Double-stainings of *in situ* hybridization and immunohistochemistry were used to verify the identity of the neurons expressing *Ano2*.

**Fig 1:**
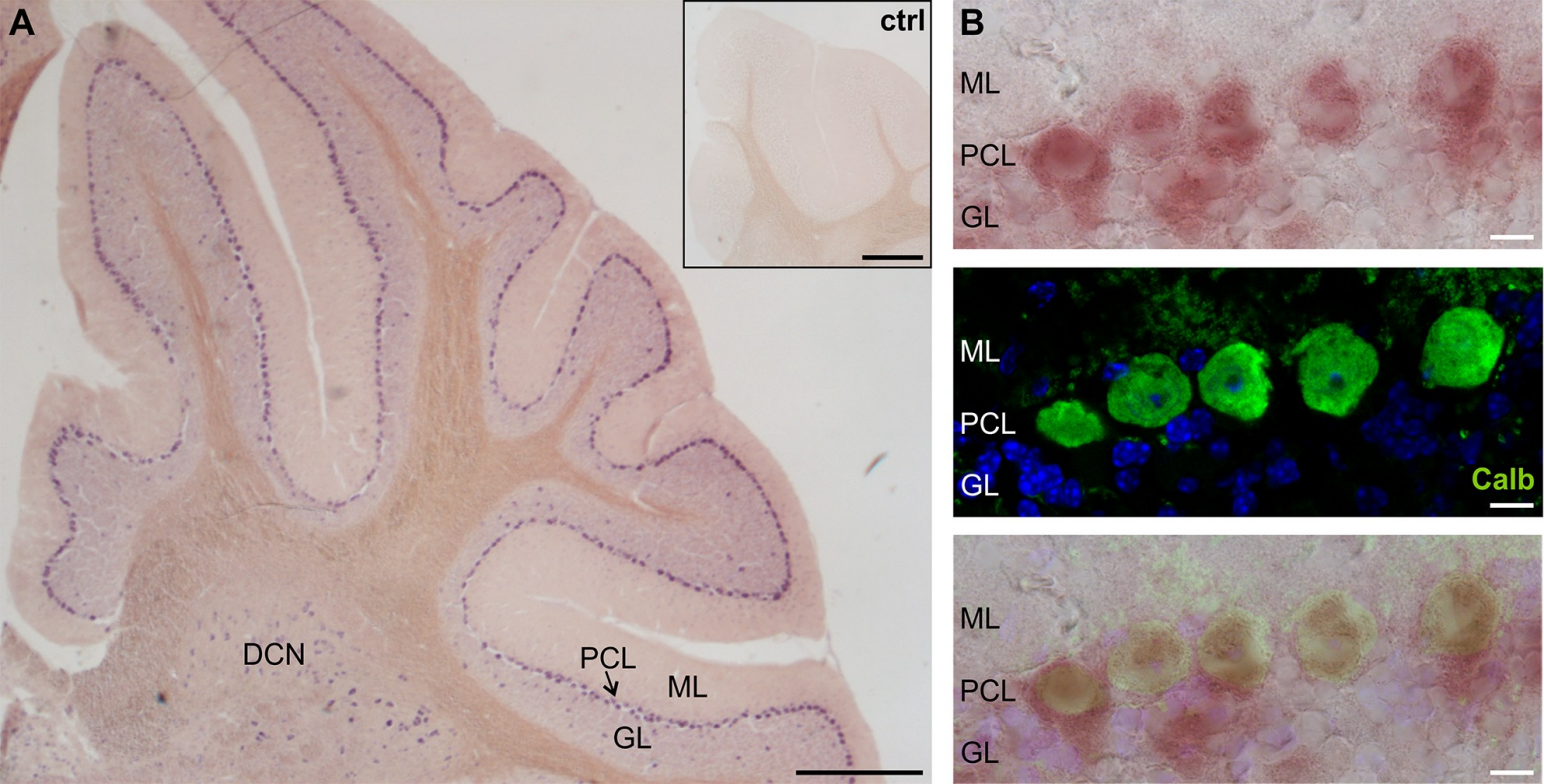
*Ano2* is expressed in cerebellar Purkinje cells. (A) Overview of *in situ* hybridization of a sagittal cerebellar slice reveals a continuous expression of *Ano2* in the Purkinje cell layer (PCL). No staining is visible in the molecular layer (ML) and only few cells are labelled in the granule layer (GL) and the area where the deep cerebellar nuclei (DCN) are located. Control experiments using the sense probes revealed no staining in the cerebellum (inset). Scale bars = 1 mm (B) Detail of the Purkinje cell layer demonstrating the colocalization (lower) of the *in situ* hybridization (upper) with an immunohistochemical staining for calbindin (green) (middle) following the *in situ* hybridization procedure. A DAPI nuclear stain (blue) was added to visualize the GL. Scale bars = 25 μm.

Our qPCR experiments revealed an expression of Ano2 mRNA in both cerebellar hemispheres and the Vermis (S1 Fig). *In situ* hybridization was performed with two different hybridization probes for *Ano2* in independent experiments. Both hybridization probes provided a robust signal along the Purkinje cell layer (PCL) in all parts of the cerebellum (Fig 1A). No signal was detectable in the control stainings with the two sense probes (Fig 1A inset). The signals for *in situ* hybridization (Fig 1B, upper) and immunohistochemistry for calbindin, a marker for Purkinje cells (Fig 1B, middle), clearly overlapped (Fig 1B, lower). These data demonstrate that *Ano2* is expressed in cerebellar Purkinje cells.

### Neuroanatomy of the cerebellar cortex is comparable in wildtype and *Ano2*^*-/-*^ mice

**Fig 2:**
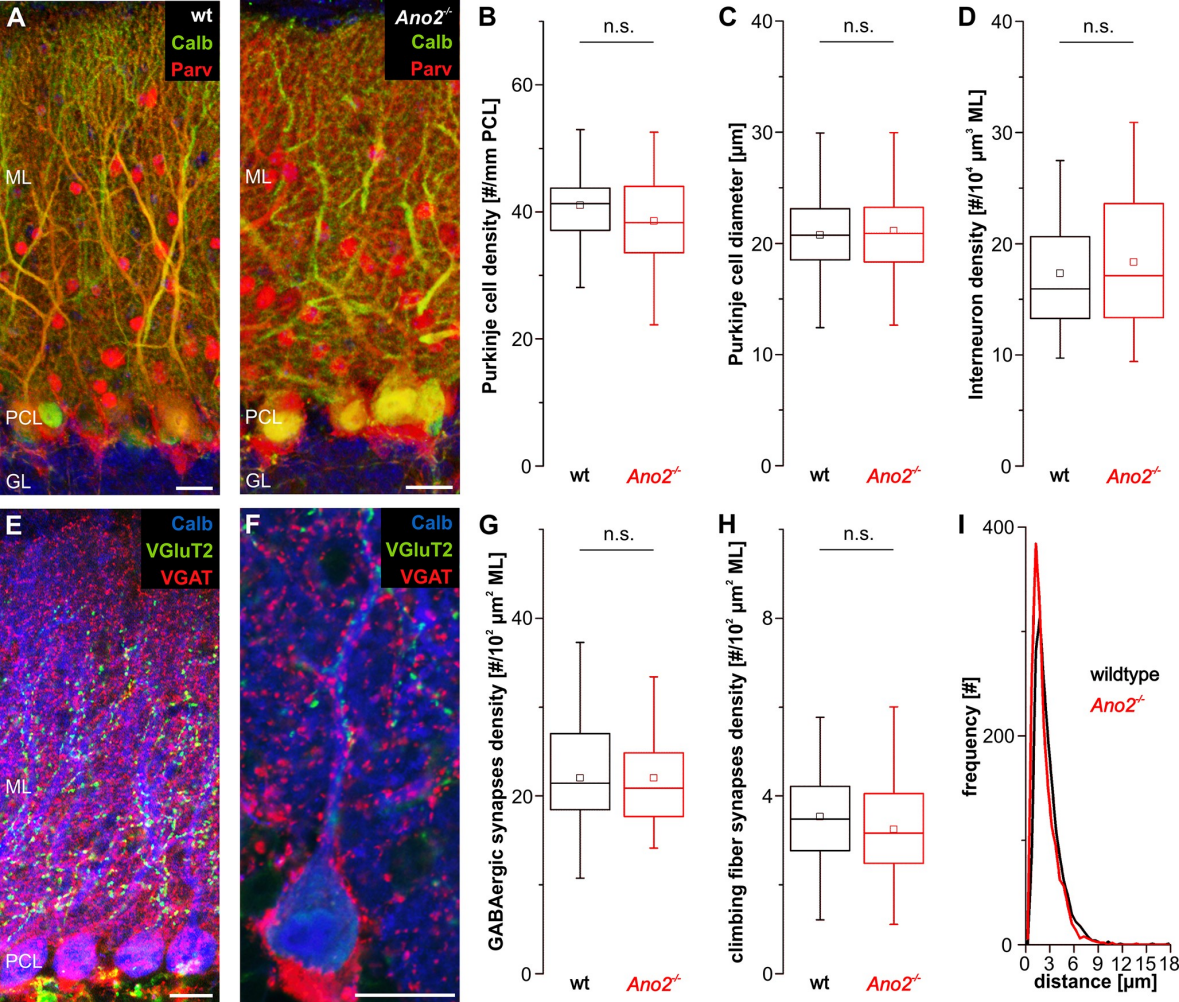
Neuroanatomy of the cerebellar cortex is comparable between wildtype and *Ano2*^-/-^ mice. (A) Representative immunohistochemical stainings for calbindin (green) and parvalbumin (red) on sagittal cerebellar slices of wildtype (left) and *Ano2*^-/-^ mice (right). Stainings were used to compare Purkinje cell density (B) and size (C) as well as the density of molecular layer interneurons (D) between wildtype and *Ano2*^-/-^ mice. DAPI (blue) was used to visualize cell nuclei. (B)–(D) No significant differences were detected in Purkinje cell density (B; wt 41.1 ± 6.4 per mm PCL, n = 61 images from N = 6 animals, *Ano2*^*-/-*^ 38.5 ± 7.5 per mm PCL, n = 42 N = 6; Student’s *t*-test *p* = 0.0811) and diameter (C; wt 20.7 ± 3.4 μm, n = 342 cells in 61 images from N = 6 animals, *Ano2*^*-/-*^ 21.1 ± 3.7 μm, n = 246 in 42 images N = 6; Student’s *t*-test *p* = 0.2091), as well as in the density of molecular layer interneurons (D; wt 15.9 per 10^5^ μm^3^ ML IQR 13.3–20.7, n = 62 images from N = 6 animals; *Ano2*^-/-^ 17.1 per 10^5^ μm^3^ ML IQR 13.3–23.6, n = 45 N = 6; Wilcoxon-Mann-Whitney test *p* = 0.501) between wildtype and *Ano2*^-/-^ mice. (E) Immunohistochemical staining for calbindin (blue), VGluT2 (green) and VGAT (red) on sagittal cerebellar slices. As expected, GABAergic synapses (red) cover the entire molecular layer, whereas climbing fiber synapses (green) only span the proximal two thirds of the molecular layer. (F) Detail of a Purkinje cell with primary and secondary dendrites (calbindin; blue) with climbing fiber synapses (VGluT2; green) and GABAergic synapses (VGAT; red). (G), (H) No significant differences were detected in density of GABAergic (G; wt 22.0 ± 6.8 per 10^2^ μm^2^ ML, n = 42 images from N = 5 animals, *Ano2*^*-/-*^ 21.8 ± 5.5 per 10^2^ μm^2^ ML, n = 47 N = 5; Student’s *t*-test *p* = 0.8788) and climbing fiber synapses (H; wt 3.5 ± 1.0 per 10^2^ μm^2^ ML, n = 42 images from N = 5 animals; *Ano2*^*-/-*^ 3.2 ± 1.0 per 10^2^ μm^2^ ML, n = 48 N = 5; Student’s *t*-test *p* = 0.1989) in the proximal half of the molecular layer between wildtype and *Ano2*^-/-^ mice. (I) Histograms of the distances measured between adjacent climbing fiber synapses on a Purkinje cell dendrite reveal a comparable distribution of climbing fiber synapses between wildtype and *Ano2*^-/-^ mice (bin size = 0.5 μm; wt n = 1698 distances in 35 images from N = 9 animals, *Ano2*^-/-^ n = 1762 in 30 images N = 6). ML = molecular layer; PCL = Purkinje cell layer; GL = granule layer. Scale bars = 20 μm.

Most experiments in this study are based on the comparison of wildtype mice and mice lacking ANO2-chloride channels (*Ano2*^-/-^ mice). To check for neuroanatomical integrity of the cerebellar cortex in *Ano2*^-/-^ mice, we analyzed density and size of Purkinje cells, as well as the density of molecular layer interneurons with double immunohistochemical stainings of the Purkinje cell marker calbindin together with parvalbumin that stains both Purkinje cells and molecular layer interneurons (Fig 2A). Additionally, we investigated the density of interneuron synapses on the Purkinje cell dendrites, as well as the density and distribution of climbing fiber synapses with co-stainings for calbindin, VGAT and VGluT2, to visualize Purkinje cells, GABAergic and climbing fiber synapses in the molecular layer of wildtype and *Ano2*^-/-^ mice, respectively (Fig 2E and 2F).

We did not observe any significant differences between wildtype and *Ano2*^-/-^ mice, neither for density (Fig 2B) or size of Purkinje cells (Fig 2C), nor for density of molecular layer interneurons (Fig 2D). As anticipated, GABAergic synapses covered the entire molecular layer, whereas climbing fiber synapses only populated the proximal two thirds of the molecular layer in both wildtype and *Ano2*^-/-^ mice. We did not detect any significant difference between wildtype and *Ano2*^-/-^ mice for the density of GABAergic synapses (Fig 2G) nor for the density of climbing fiber synapses (Fig 2H). Climbing fiber synapses were regularly distributed in both wildtype and *Ano2*^-/-^ mice, with distances typically ranging from 1 to 5 μm, without clusters of synapses being observable (Fig 2I).

These results provide evidence, that the neuroanatomical parameters of the cerebellar cortex of *Ano2*^-/-^ mice are not significantly altered compared to wildtype mice. Therefore, we can assume that functional differences between wildtype and *Ano2*^-/-^ mice are due to the absence of ANO2 chloride-channels as opposed to being a consequence of neuroanatomical changes in the cerebellar cortex of *Ano2*^-/-^ mice.

### ANO2 does not affect spontaneous simple spike activity of Purkinje cells

Purkinje cells display intrinsically driven simple spike discharge at high rates (ca. 40–50 Hz) which is modulated by parallel fiber input [34,35]. Additionally, Purkinje cells receive excitatory input from climbing fibers that irregularly elicit complex spikes in Purkinje cells at low rates (ca. 1 Hz) [36]. We performed current-clamp measurements on Purkinje cells in acute cerebellar slices to analyze spontaneous simple spike discharge of Purkinje cells in wildtype and *Ano2*^-/-^ mice. To monitor this spontaneous activity, Purkinje cells were held at 0 pA.

**Fig 3:**
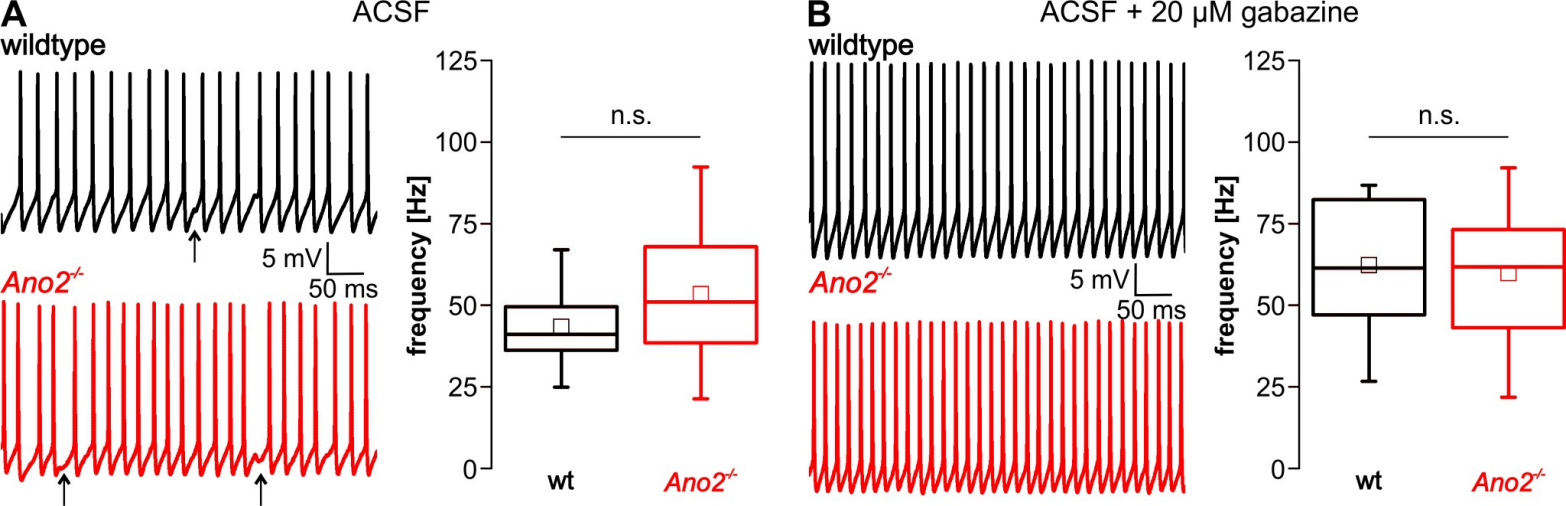
Spontaneous simple spike firing of Purkinje cells is unaltered in *Ano2*^-/-^ mice. Representative traces of spontaneous simple spike activity in wildtype (black) and *Ano2*^-/-^ mice (red) before (A) and after (B) addition of 20 μM gabazine to the bath solution. The arrows in (A) mark spontaneous inhibitory postsynaptic currents (IPSCs) that were then blocked by addition of gabazine to the bath solution. Comparison of spontaneous simple spike firing rates reveals no significant difference between wildtype and *Ano2*^-/-^ mice without (A; wt 43.3 ± 12.9 Hz, n = 10 cells of N = 3 animals; *Ano2*^-/-^ 53.2 ± 21.4 Hz, n = 15 N = 3; Student’s *t*-test *p* = 0.1631) and with 20 μM gabazine (B; wt 62.3 ± 21.5 Hz, n = 12 cells of N = 3 animals; *Ano2*^-/-^ 59.8 ± 20.4 Hz, n = 23 N = 5; Student’s *t*-test *p* = 0.7448).

The measured spontaneous simple spike firing rates of wildtype mice without gabazine corresponded to published data [35] and no significant difference was observed between wildtype and *Ano2*^-/-^ mice (Fig 3A). We added 20 μM gabazine to the bath solution to eliminate chloride currents conducted by GABA_A_-mediated channels. As expected, spontaneous simple spike activity became more regular and firing rates increased but were still comparable between wildtype and *Ano2*^-/-^ mice (Fig 3B). These results suggest, that ANO2 does not modulate spontaneous simple spike discharge of Purkinje cells.

### ANO2-mediated chloride currents reduce excitability of Purkinje cells

Spontaneous simple spike activity of Purkinje cells can be modulated by inhibitory and excitatory input from molecular layer interneurons and parallel fibers, respectively, leading to simple spike rates of up to 250 Hz [37,38]. To investigate the excitability of Purkinje cells, we first applied hyperpolarizing currents to clamp Purkinje cells close to -70 mV and then injected depolarizing current pulses of 200 to 1000 pA amplitude. Current injection of 200 pA resulted in threshold activation [39], 600 pA in moderate activation and 1000 pA in strong activation of Purkinje cells. Simple spikes were counted in the first 500 ms of each current pulse. Additionally, spike frequency adaptation as well as the hyperpolarization following the current injections were analyzed. Because ANO2-mediated changes in excitability could either be caused by chloride currents or, alternatively, by a shunt conductance via ANO2, the recordings were repeated with a slightly modified intracellular solution, where the chloride concentration was adjusted to raise the reversal potential for chloride currents (E_Cl_) from -87 mV to -48 mV. As -48 mV corresponded to the mean simple spike threshold (S2D Fig), this approach resulted in a minimized net chloride current at the simple spike threshold, whereas the shunt conductance was still effective. Therefore, this second set of experiments allowed to distinguish between effects that were caused by chloride currents via ANO2 or by an ANO2-mediated shunt conductance. All recordings were performed in the presence of 20 μM gabazine, to eliminate GABA_A_-mediated chloride currents.

**Fig 4:**
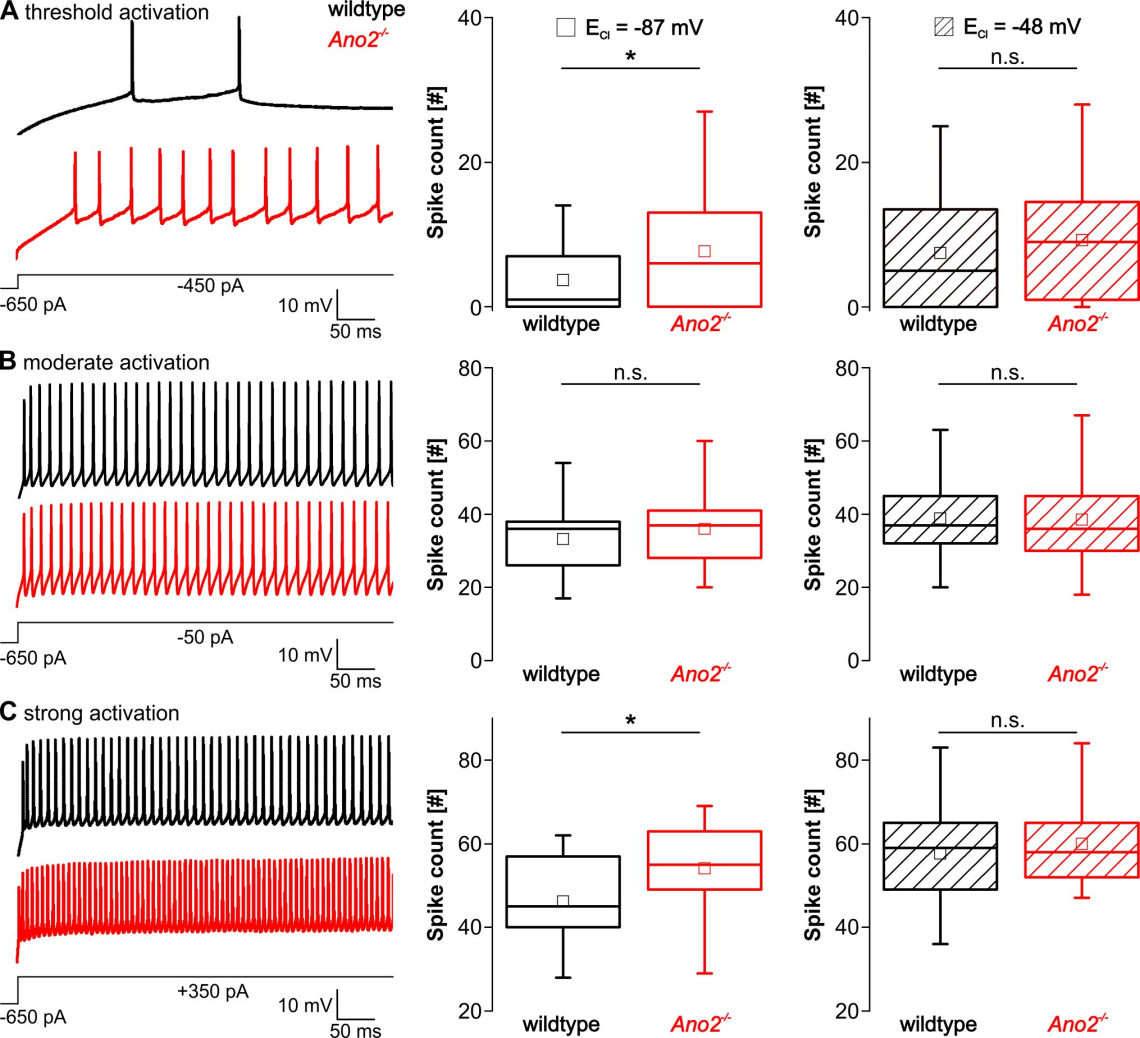
ANO2 reduces excitability during strong and threshold activation. Analysis of simple spike counts upon threshold activation (A), moderate activation (B) and strong activation (C). Representative traces (left) of simple spike activity of Purkinje cells from wildtype (black) and *Ano2*^-/-^ mice (red) at physiological E_Cl_. Comparison of simple spike counts during the first 500 ms of the respective current injection at physiological (middle) and elevated E_Cl_ (right) (A middle: wt 1.0 IQR 0.0–7.0, n = 39 cells of N = 8 animals, *Ano2*^-/-^ 6.0 IQR 0.0–13.0, n = 43 cells of N = 11 animals, Wilcoxon-Mann-Whitney test *p* = 0.040; A right: wt 5.0 IQR 0.0–14.3, n = 32 cells of N = 6 animals, *Ano2*^-/-^ 9.0 IQR 1.0–14.75, n = 36 cells of N = 8 animals, Wilcoxon-Mann-Whitney test *p* = 0.368; B middle: wt 33.1 ± 11.0, n = 35 cells of N = 8 animals, *Ano2*^-/-^ 35.9 ± 11.8, n = 31 N = 9, Student’s *t*-test *p* = 0.3255; B right: wt 38.9 ± 11.1, n = 29 cells of N = 6 animals, *Ano2*^-/-^ 38.5 ± 10.6, n = 31 N = 8, Student’s *t*-test *p* = 0.9116; C middle: wt 46.1 ± 11.0, n = 21 cells of N = 6 animals, *Ano2*^-/-^ 54.1 ± 10.6, n = 19 N = 7, Student’s *t*-test *p* = 0.0255; C right: wt 57.6 ± 11.4, n = 27 cells of N = 6 animals, *Ano2*^-/-^ 60.0 ± 13.1, n = 29 N = 7, Student’s *t*-test *p* = 0.4788). * *p* < 0.05.

As expected, threshold activation of Purkinje cells of wildtype mice induced only a few simple spikes or no spikes at all (Fig 4A left). In contrast, repetitive simple spike activity could regularly be observed in Purkinje cells of *Ano2*^-/-^ mice upon the same current injection intensity. This resulted in a significantly increased average number of simple spikes upon threshold activation in *Ano2*^-/-^ mice (Fig 4A middle). Repetitive simple spike activity was also significantly increased in *Ano2*^-/-^ mice upon strong current injections (Fig 4C), whereas only a slight tendency towards increased simple spike activity could be observed in *Ano2*^-/-^ mice upon moderate current injections (Fig 4B). Interestingly, these differences between wildtype and *Ano2*^-/-^ mice disappeared upon elevation of E_Cl_ to the simple spike threshold. Moreover, the elevation of E_Cl_ appeared to only affect the simple spike activity of wildtype but not *Ano2*^-/-^ Purkinje cells. The elevation of E_Cl_ neither influenced the basic electrical properties of Purkinje cells, such as input resistance and simple spike threshold, nor the spontaneous simple spike activity (S2 Fig).

**Fig 5:**
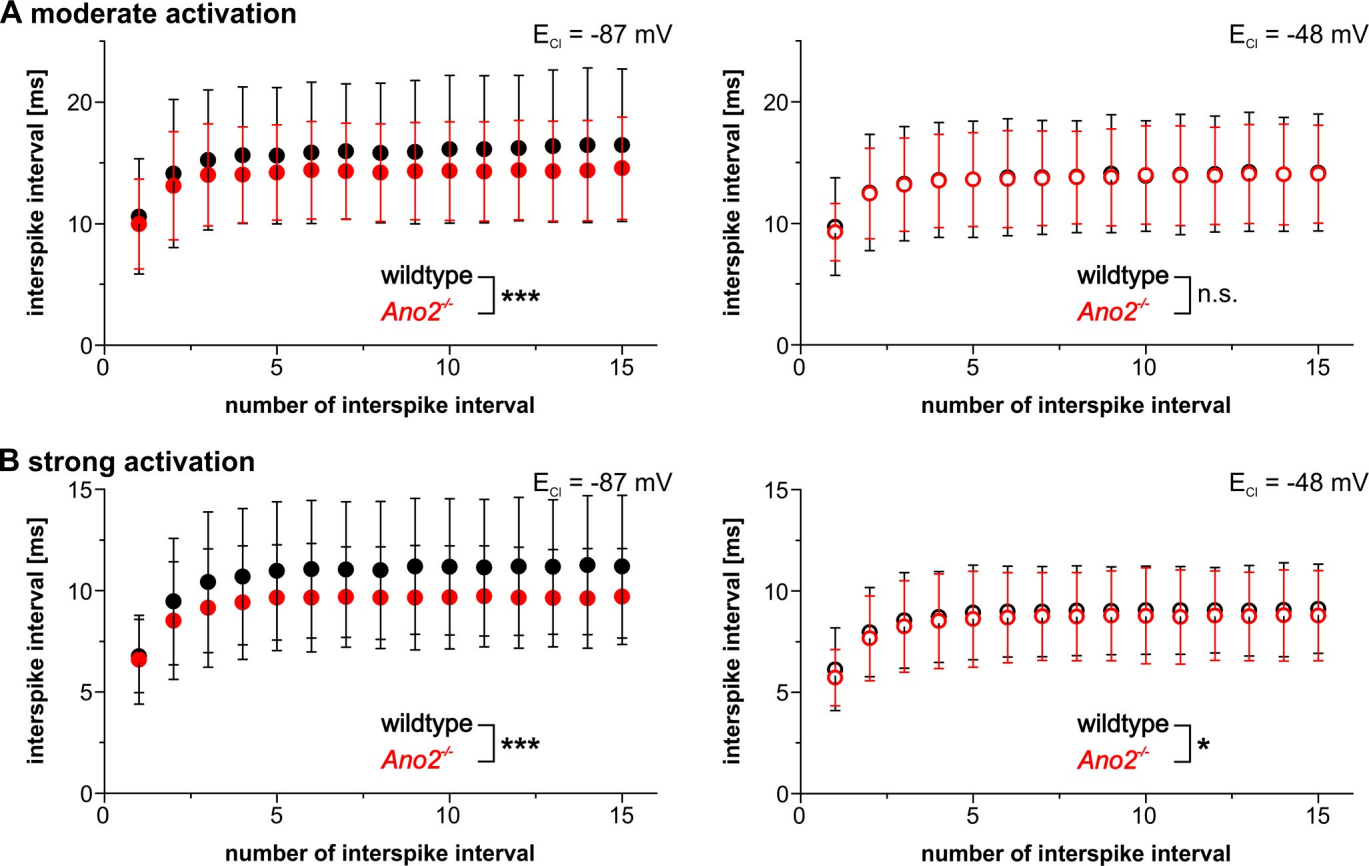
ANO2-mediated chloride currents gradually increase interspike intervals. Progression of interspike intervals (ISIs) during the first sixteen simple spikes upon moderate (A) and strong activation (B) of Purkinje cells in wildtype (black) and *Ano2*^-/-^ mice (red). At physiological E_Cl_ (left), the interspike intervals in wildtype mice grow longer from the second ISI on, causing a divergence of the ISIs from wildtype and *Ano2*^-/-^ mice. In contrast, the progression of ISIs at elevated E_Cl_ (right) develops without divergence (A left: Genotype effect: F(1, 930) = 22.65, *p* < 0.001; ISI number x genotype interaction: F(14, 930) = 0.09, *p* = 1; A right: Genotype effect: F(1, 900) = 0.18, *p* = 0.6712; ISI number x genotype interaction: F(14, 930) = 0.01, *p* = 1; B left: Genotype effect: F(1, 570) = 29.37, *p* < 0.001; ISI number x genotype interaction: F(14, 570) = 0.14, *p* = 1; B right: Genotype effect: F(1, 840) = 4.2, *p* = 0.0430; ISI number x genotype interaction: F(14, 840) = 3.7, *p* = 1). * *p* < 0.05; *** *p* < 0.001.

**Fig 6:**
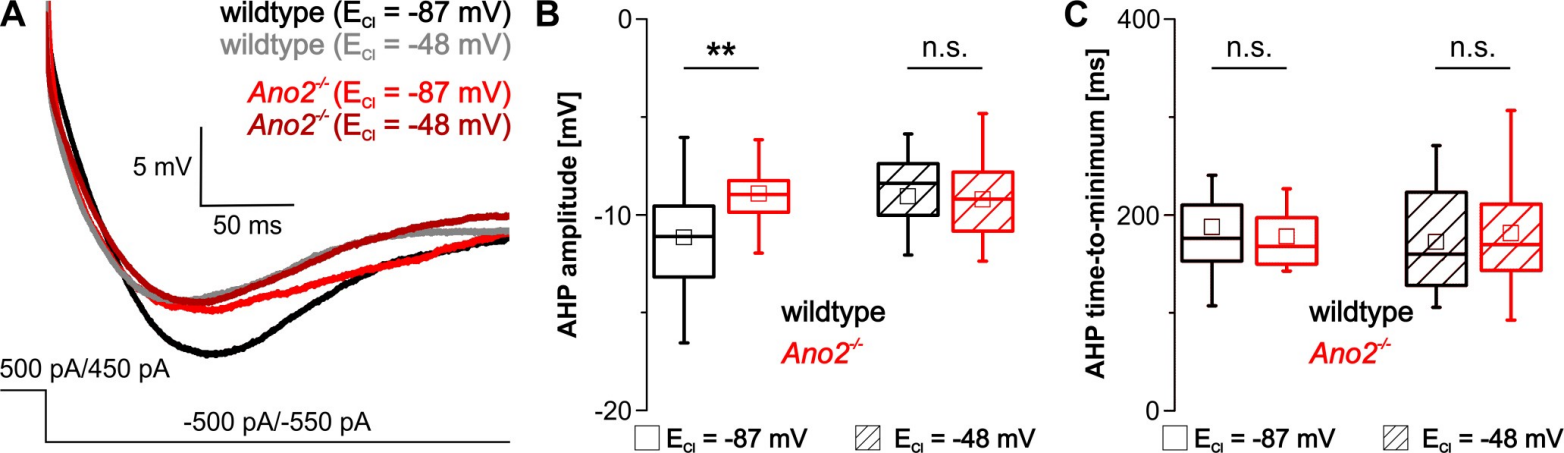
ANO2 increases the hyperpolarization following strong activation of Purkinje cells. (A) Representative traces of the afterhyperpolarization (AHP) following strong activation of wildtype (black/grey) and *Ano2*^-/-^ (red/dark red) Purkinje cells at physiological and elevated E_Cl_. The holding current was set at -500 pA or -550 pA for all cells in this example. (B) AHP amplitude after strong activation is significantly larger in wildtype compared to and *Ano2*^-/-^ mice at physiological E_Cl_, but not at elevated E_Cl_ (E_Cl_ -87 mV: wt -11.2 ± 2.8 mV, n = 20 cells of N = 6 animals; *Ano2*^*-/-*^ -8.9 ± 2.4 mV, n = 19 N = 7; Student’s t-test *p* = 0.0097; E_Cl_ -48 mV: wt -9.0 ± 3.0 mV, n = 27 cells of N = 6 animals; *Ano2*^*-/-*^ -9.2 ± 1.9 mV, n = 31 N = 7; Student’s t-test *p* = 0.7983) (C) Time till the maximal AHP is reached after strong activation is unaltered by ANO2 or the elevation of E_Cl_ (E_Cl_ -87 mV: wt 187.8 ± 53.2 ms, n = 20 cells of N = 6 animals; *Ano2*^*-/-*^ 178.1 ± 35.6 ms, n = 19 N = 7; Student’s t-test *p* = 0.5073; E_Cl_ -48 mV: wt 172.4 ± 50.7 mV, n = 27 cells of N = 6 animals; *Ano2*^*-/-*^ 181.4 ± 49.8 ms, n = 31 N = 7; Student’s t-test *p* = 0.5035). ** *p* < 0.01.

Spike frequency adaptation was examined by analyzing the progression of interspike intervals (ISIs) for moderate and strong current injections. Because threshold activation did often induce only few simple spikes, spike frequency adaptation was not analyzed in this case. At physiological E_Cl_, the first ISIs were similar in wildtype and *Ano2*^-/-^ mice for both moderate and strong activation but then gradually diverged, with ISIs growing larger in wildtype compared to *Ano2*^-/-^ Purkinje cells (Fig 5 left). In contrast, when the driving force for chloride was reduced no divergence of ISIs was observed for both moderate and strong current injections (Fig 5 right). Only a minimal parallel shift between wildtype and *Ano2*^-/-^ mice were observed upon strong activation of Purkinje cells. Furthermore, we analyzed the extent and time course of hyperpolarization that occurred after returning to the holding current that clamped Purkinje cells close to -70 mV. The amplitude of afterhyperpolarization was significantly decreased in *Ano2*^-/-^ mice compared to wildtype following strong activation at physiological, but not elevated E_Cl_ (Fig 6B). The same tendency, although not statistically significant, was observed after moderate and threshold activation (S3 Fig). No difference between wildtype and *Ano2*^-/-^ mice was observed for the time until maximal afterhyperpolarization, neither at physiological nor at increased intracellular chloride concentration.

Taken together, these results indicate that ANO2-mediated chloride currents reduce the excitability of Purkinje cells at the activation threshold and during strong activation. These chloride currents lead to a prolongation of ISIs from the second simple spike on and additionally promote hyperpolarization after current injections stop.

### ANO2 attenuates inhibition after depolarization of Purkinje cells

It has previously been suggested that ANO2 is involved in a process causing attenuation of GABAergic inhibition following climbing fiber input [10]. This process was proposed to rely on a calcium-mediated influx of chloride into the dendrites that increases the local intracellular chloride concentration and, thus, diminishes the driving force for chloride across the dendritic membrane [12]. The resulting reduction of amplitude observed in spontaneous inhibitory postsynaptic currents (IPSCs) at GABAergic synapses of wildtype mice, could not be detected in *Ano2*^-/-^ mice [10]. This finding gave rise to the idea that ANO2 would temporarily relieve Purkinje cells from GABAergic inhibition after a calcium surge that accompanies a complex spike. However, it is still unclear whether this effect is based on ANO2 channels in inferior olivary neurons or ANO2 channels in the dendritic membrane of Purkinje cells.

**Fig 7:**
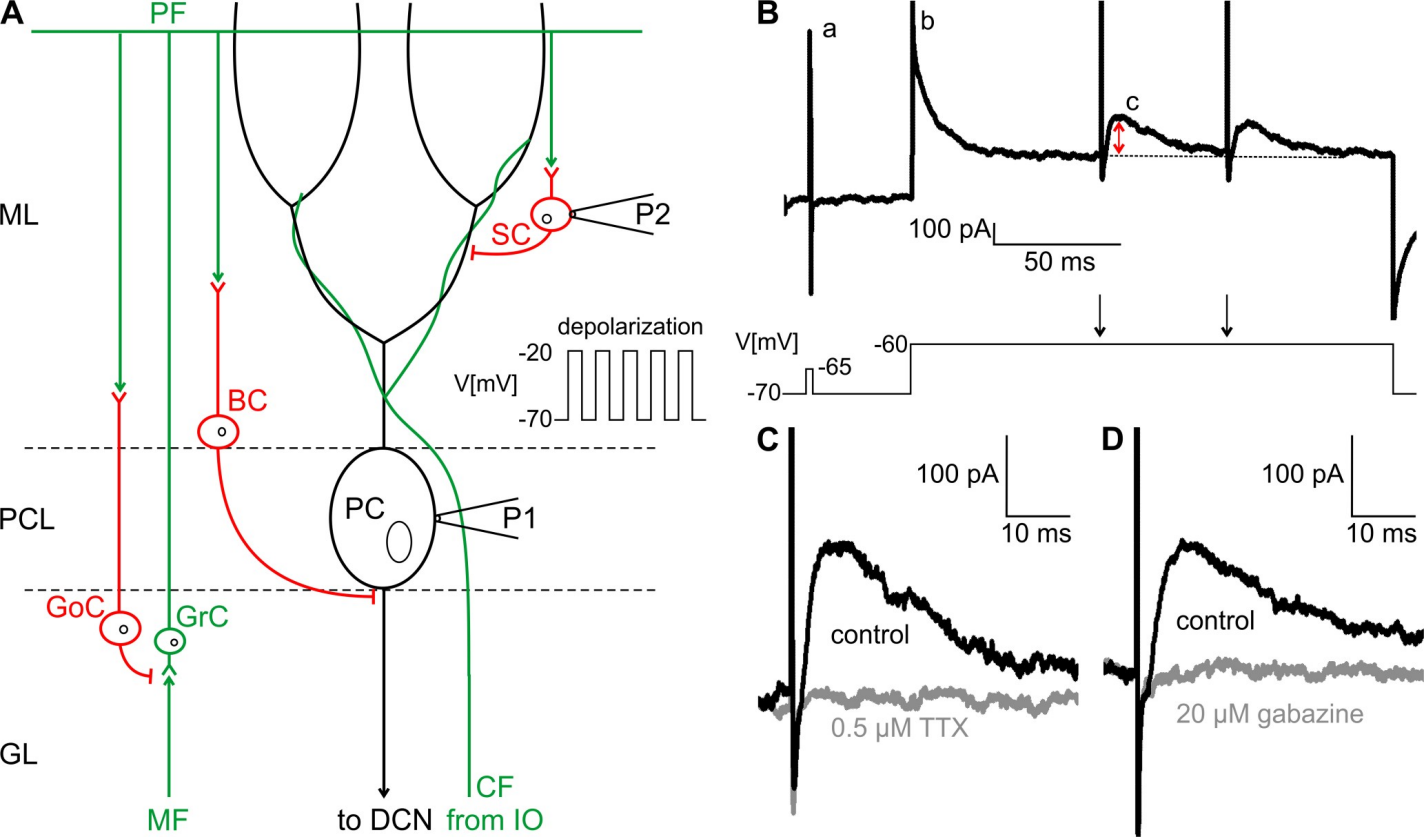
Stimulation of molecular layer interneurons and control experiments. (A) Schematic illustration of the neuronal circuits in the cerebellar cortex and the protocol for the stimulation of molecular layer interneurons. Purkinje cells (PC) are the central element in the olivo-cerebellar circuit. Purkinje cells project to the deep cerebellar nuclei (DCN) and receive excitatory input from the mossy fiber (MF)-parallel fiber (PF) pathway and from climbing fibers (CF), which originate in the inferior olivary nucleus (IO) of the brainstem. Basket cells (BC) and stellate cells (SC) both inhibit Purkinje cells, whereas Golgi cells (GoC) inhibit the mossy fiber-granule cell (GrC) synapse. Evoked inhibitory postsynaptic currents (eIPSCs) are induced by extracellular stimulation of molecular layer interneurons (P2). eIPSCs are recorded at the Purkinje cell soma with the patch pipette (P1). Reduction of eIPSC amplitudes is induced by a train of five depolarizing pulses with an amplitude of 50 mV via the patch pipette (inset). GL = granule layer, PCL = Purkinje cell layer, ML = molecular layer (B) Representative trace of an eIPSC recording described in (A). At the beginning of each recording, a test pulse (a) was applied to monitor series resistance. The holding potential was increased to -60.5 mV (b) before inducing two eIPSCs (c) with an interstimulus interval of 50 ms (arrows in the command trace). The red arrow marks the amplitude of eIPSC that was analyzed in these experiments (C), (D) Representative traces of eIPSC recordings of one cell before (black) and after (grey) adding 0.5 μM TTX (C) or 20 μM gabazine (D) to the bath solution. eIPSCs are completely abolished by both TTX and gabazine.

To address this question, we performed whole-cell voltage clamp recordings on Purkinje cells uncoupled from climbing fiber input, using a stimulation protocol slightly modified from [12] (Fig 7A). In contrast to the previously performed experiments [10], IPSCs were evoked by stimulation of individual molecular layer interneurons with an ACSF-filled glass pipette positioned in the molecular layer (P2 in Fig 7A). Purkinje cells were held at a membrane potential of -70.5 mV, which was increased to -60.5 mV during stimulation of molecular layer interneurons in order to provide a better signal-to-noise ratio of the evoked IPSCs (eIPSCs; b in Fig 7B). Molecular layer interneurons were stimulated twice (arrows in Fig 7B) with an interstimulus interval of 50 ms to monitor the paired-pulse ratio as a parameter for recording stability and postsynaptic localization of the effect. A pipette solution with low chloride was used in all recordings resulting in an E_Cl_ of -87 mV. Therefore, chloride currents, such as eIPSCs, appeared as outward currents in the recordings (c in Fig 7B). Reduction of eIPSC amplitude was induced, as previously described [12], with five depolarizing pulses to -20.5 mV (inset in Fig 7A) via the patch pipette (P1 in Fig 7A). Each depolarization step lasted for 1 s and was separated from the next depolarization step by another 1 s (0.5 Hz). Glutamatergic inputs as well as CB_1_ and GABA_B_ mediated signals were blocked by bath application of CNQX (10 μM), D-AP5 (40 μM), AM 251 (2 μM) and CGP 55845 (2 μM) in all recordings. eIPSCs were completely abolished in the presence of 0.5 μM TTX (Fig 7C) or 20 μM gabazine (Fig 7D). The polarity of the currents together with their sensitivity for TTX and gabazine indicates, that the evoked IPSCs are indeed mediated by molecular layer interneurons.

**Fig 8:**
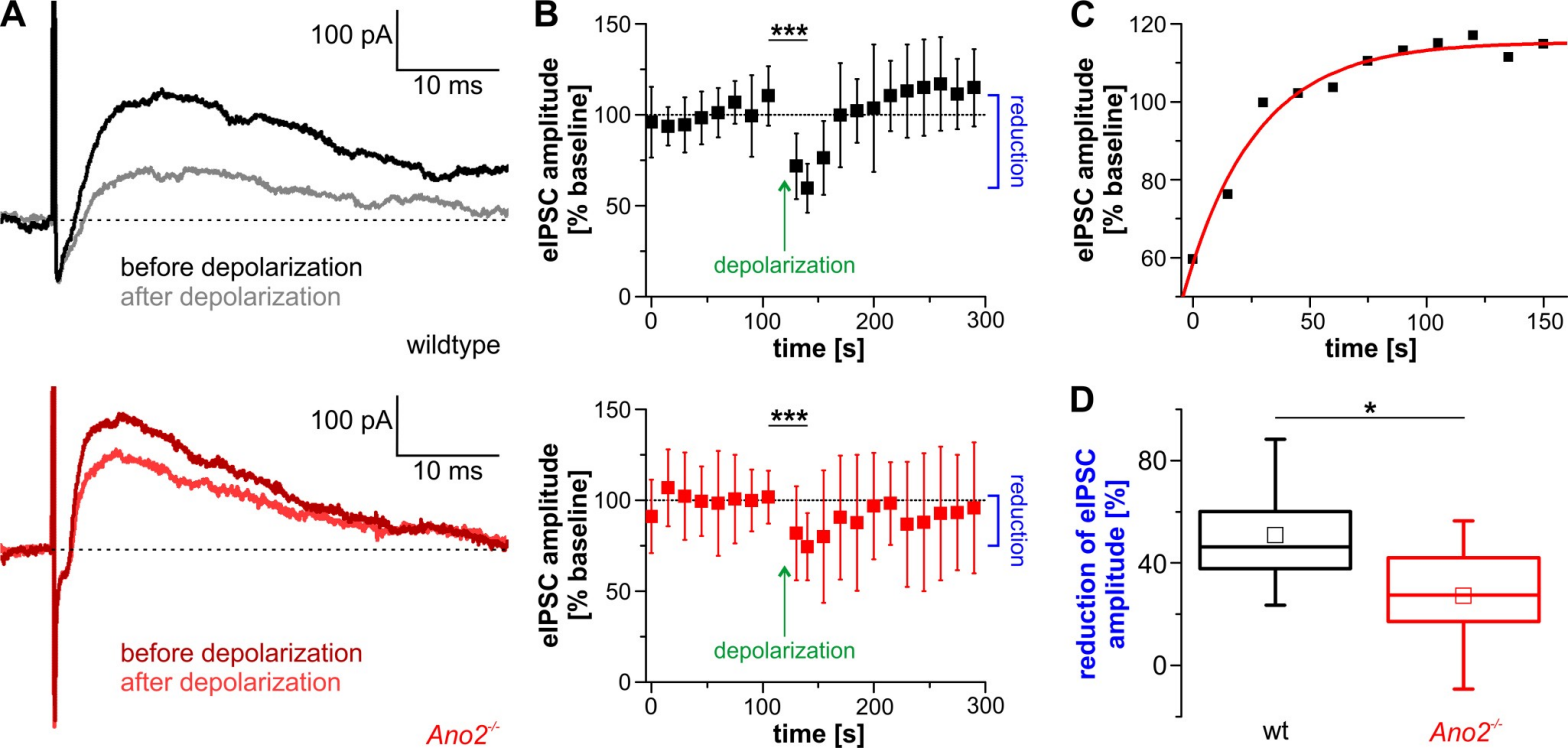
ANO2 promotes reduction of eIPSC amplitude. (A) Representative traces of eIPSC recordings in wildtype (upper) and *Ano2*^-/-^ mice (lower) before (black/dark red) and after depolarization (grey/light red) of Purkinje cells. The dotted line marks the holding current right before stimulation of interneurons from which the amplitude was calculated. (B) Time course of eIPSC amplitude in wildtype (upper) and *Ano2*^-/-^ mice (lower). Reduction (blue) was defined as the difference between eIPSC amplitude of the last measurement before depolarization and eIPSC amplitude of the second measurement after depolarization. eIPSC amplitude was significantly reduced in both mouse lines after depolarization (wt before depol. 110.4 ± 16.3%, wt after depol. 59.6 ± 13.4%, paired Student’s *t*-test *p* = 0.0001; *Ano2*^-/-^ before depol. 101.6 ± 14.5%, *Ano2*^-/-^ after depol. 74.5 ± 18.4%, paired Student’s *t*-test *p* = 0.0004). (C) Recovery of eIPSC amplitude in wildtype Purkinje cells after depolarization (B, upper). An exponential fit (red) to the mean values of eIPSC amplitude was performed to obtain the time constant τ = 30.6 s of recovery. (D) Comparison of reduction of eIPSC amplitudes (blue in B) between wildtype and *Ano2*^-/-^mice. The reduction of eIPSC amplitude is significantly attenuated in *Ano2*^-/-^ mice (wt 50.8 ± 20.9%, *Ano2*^-/-^ 27.2 ± 21.2%, Student’s *t*-test *p* = 0.0173). wt n = 9 cells from N = 6 animals; *Ano2*^-/-^ n = 14, N = 9; * *p* < 0.05, *** *p* < 0.001.

To investigate whether an ANO2-mediated chloride current attenuates inhibition after depolarization of Purkinje cells, we measured the eIPSC amplitude (red arrow in Fig 7B) every 15 s before and after depolarizing Purkinje cells of wildtype and *Ano2*^-/-^ mice via the patch pipette (Fig 8A). To compare recordings of different cells, eIPSC amplitudes were normalized to the averaged eIPSC amplitude preceeding depolarization (baseline). eIPSC amplitudes were significantly reduced in wildtype mice 15 s after depolarization (Fig 8B, upper). In accordance with previous experiments [12], paired-pulse ratios did not change after depolarization (S4 Fig). We observed a complete recovery of eIPSC amplitude with time constant τ = 30.6 s, which was calculated by performing a monoexponential fit to the mean values of eIPSC amplitudes (Fig 8C). In *Ano2*^-/-^ mice, the eIPSC amplitude was also reduced significantly after depolarization of Purkinje cells (Fig 8B, lower). Comparing both datasets revealed that the effect of depolarization on the eIPSC amplitudes was significantly stronger in wildtype compared to *Ano2*^-/-^ mice (Fig 8D).

The observed reduction of eIPSC amplitudes in wildtype mice occurred without contribution of climbing fibers. Because this reduction was diminished in *Ano2*^-/-^ mice, we conclude that ANO2 in Purkinje cells must contribute to this process, although other factors appear to be involved as well.

Taken together, our results provide conclusive evidence that ANO2 is expressed in cerebellar Purkinje cells where it reduces simple spike activity during strong activation and at the activation threshold. Additionally, ANO2 seems to be activated upon strong depolarization in the dendrites, which leads to a transient reduction of IPSC amplitudes.

## Discussion

One of the main underlying questions of the controversy regarding the role of ANO2 in the olivo-cerebellar system is the exact location of expression of ANO2. As many other ion channels in the brain, ANO2 channels are expressed at low density and, therefore, sensitive methods are needed for their detection [3,6]. Initial evidence for the expression of ANO2 in Purkinje cells has previously been provided with immunohistochemical studies [10,11]. Moreover, with electrophysiological recordings using niflumic acid as a blocker [12] as well as with the Anoctamin-specific blocker T16Ainh-A01 [10], a calcium-dependent chloride current was identified in Purkinje cells. Consistent with these data, we detected the expression of *Ano2* mRNA in cerebellar tissue using quantitative real-time PCR (see S1 Fig). These results indicate a more widespread expression *Ano2* in the olivo-cerebellar system, not restricted to the inferior olivary nucleus, which has already been established as an expression site of *Ano2* [9]. With our *in situ* hybridization experiments, performed with two different hybridization probes and co-stained with an antibody against the Purkinje cell-specific marker calbindin, we have clearly identified Purkinje cells to express *Ano2*. Our findings may seem to contradict recent findings obtained from the *Ano2*-knockout mouse line expressing farnesylated mCherry instead of ANO2 [9]. No mCherry fluorescence was detected in Purkinje cells of this mouse model [9]. Such a discrepancy also occurred when the expression of *Ano2* in somatostatin-positive GABAergic neurons of the central lateral amygdala was described [6]. This study also used an *in situ* hybridization approach to detect an expression of *Ano2*, although the mCherry-expressing *Ano2*-knockout mouse line was used for the electrophysiological recordings [6]. This indicates that the mCherry fluorescence might not be strong enough to reliably reveal an expression of *Ano2* at low density.

Thus, the current evidence suggests that *Ano2* is expressed in two distinct interconnected types of neurons in the olivo-cerebellar system: inferior olivary neurons and Purkinje cells.

In the present study, we monitored changes in excitability of Purkinje cells by analyzing simple spike activity in acute cerebellar slices. To examine the contribution of ANO2-chloride channels, we compared recordings of wildtype to *Ano2*^*-/-*^ mice. We did not observe any complex spikes in our recordings, excluding any impact on our results of altered climbing fiber signaling due to the loss of ANO2 in inferior olivary neurons. While spontaneous simple spike discharge was unaltered in *Ano2*^*-/-*^ mice, we detected an increased simple spike rate upon threshold activation and during high simple spike activity. These results are supported by the observation that wildtype Purkinje cells develop prolonged interspike intervals compared to *Ano2*^*-/-*^ mice, although the initial interspike intervals following current injection are similar. We demonstrated that a chloride current via ANO2 is the main cause of the observed dampening of excitability, as differences in excitability between wildtype and *Ano2*^*-/-*^ mice disappeared when the net chloride driving force was minimized. Nevertheless, a small but constant prolongation of the interspike intervals could be observed in wildtype mice following strong activation of Purkinje cells, when the driving force for chloride was reduced. Therefore, a small contribution of a shunt conductance via ANO2 cannot be excluded. The hyperpolarizing impact of ANO2-mediated chloride currents appears to increase the activation threshold of Purkinje cells in wildtype mice. We conclude that ANO2 channels have a certain open probability, even at membrane potentials close to -70 mV, despite their comparatively low sensitivity towards calcium (half-maximally activation at 1.33 μM Ca^2+^ [40]) and the high endogenous buffering capacity for calcium of Purkinje cells [41]. We hypothesize that ANO2-chloride channels are located in close proximity to sites of calcium entry, such as voltage-gated calcium channels, as it has been suggested for ANO2 in the hippocampus and the inferior olivary nucleus [3,9]. In the sensitive state of threshold activation even small hyperpolarizing chloride currents via ANO2 seems to be sufficient to significantly lower the activation threshold.

It was previously shown that simple spike activity of Purkinje cells leads to a rise of the intracellular calcium concentration that is largely restricted to the soma [42,43]. Furthermore, the expression of ANO2-chloride channels in the soma of Purkinje cells has previously been suggested with immunohistochemical studies [10,11]. Consequently, increasing somatic calcium concentrations may further activate ANO2 channels in the somatic plasma membrane, thus strengthening the hyperpolarizing chloride current. This chloride current causes only a minimal reduction in simple spike activity during moderate activation, which causes repetitive simple spike activity. Moderate activation in our experiments resembles the state of spontaneous activity (40–50 Hz), dominated by several voltage-gated ion channels. This state is not significantly influenced by the comparatively small chloride current via ANO2 that is induced during moderate activation. With increasing somatic calcium concentrations during stronger activation, the inhibitory effect of ANO2-mediated chloride currents increases and causes a significantly reduced simple spike activity upon strong activation in wildtype mice. In this condition, ANO2-chloride channels are activated more strongly because of the increase of the intracellular calcium concentration and the fact that the channels’ sensitivity towards calcium increases with depolarization [44,45]. Thus, our data consistently demonstrate an activity-dependent inhibitory influence of ANO2 channels. Additionally, chloride influx via ANO2 increases the afterhyperpolarization upon return to a membrane potential close to -70 mV. Thus, our results are consistent with the hypothesis that ANO2 effects critically depend on the intracellular calcium concentration. While low calcium levels produce an inhibitory effect only near the activation threshold of simple spikes, elevated calcium levels exert a measurable ANO2-mediated effect during strong activation.

Immunohistochemical stainings indicated the expression of ANO2 in the Purkinje cell soma and in the dendritic tree up to the third order branching [10]. A possible expression of ANO2 protein in the fine distal dendrites still needs to be determined. This raises the question as to whether ANO2 is also active when the dendrites are loaded with calcium, which occurs for example after climbing fiber input [43] that triggers dendritic calcium influx [46,47]. Previous studies presented evidence for ANO2-mediated disinhibition following climbing fiber stimulation [10,12]. This effect developed markedly slower than the attenuation of simple spike activity and was proposed to result from chloride accumulation within the dendrite. To identify the role of the intrinsic Purkinje cell ANO2 channels in this disinhibition, we induced calcium influx through somatic depolarization of Purkinje cells via the patch pipette instead via stimulation of climbing fibers. We stimulated molecular layer interneurons in regular intervals to monitor the inhibitory input over time. We observed a reduction of inhibitory postsynaptic currents for several seconds after depolarization of Purkinje cells and revealed a significant contribution of ANO2 to this effect. Therefore, we conclude that ANO2 is also activated in the dendrites and causes local influx of chloride at elevated calcium concentrations. This hyperpolarizing chloride current is accompanied by an accumulation of chloride that has been suggested to reduce the driving force for chloride over the dendritic membrane and, hence, to reduce inhibitory inputs [12]. It still remains to be clarified whether this reduction of inhibition is physiologically relevant or represents a mere side effect of the hyperpolarizing chloride current via ANO2.

In a previous study, hints on an additional expression of ANO1 calcium-activated chloride channels in the soma of Purkinje cells and other GABAergic neurons in the cerebellar cortex were provided using immunohistochemical stainings [10]. The function of these channels in the cerebellum still needs to be determined. A contribution of ANO1 to fast processes such as modulation of spike frequency adaptation of simple spikes appears, however, unlikely due to their activation kinetics in the range of 100 to 300 ms, about ten times slower than the activation of ANO2 [48]. An involvement of ANO1 in the reduction of IPSC amplitude following climbing fiber stimulation, which occurs on much longer time scales, was ruled out using T16Ainh-A01 at a concentration of 5 μM, which causes a reduction of the chloride current via ANO2, but not ANO1 [10]. In the presence of T16Ainh-A01, no reduction of IPSC amplitude could be observed.

The data presented in this study, together with the previously reported evidence for impaired signaling of inferior olivary neurons in *Ano2*-knockout mice [9], suggest a complex modulation of signal processing in the olivo-cerebellar system via ANO2-chloride channels. In inferior olivary neurons, ANO2 facilitates fast spike generation [9] whereas, in Purkinje cells, ANO2 increases the activation threshold and reduces simple spike activity upon strong activation. This could serve as a mechanism of self-inhibition as it has been reported for ANO2 in fast-spiking thalamocortical neurons [4]. The hyperpolarizing chloride current that follows activation of ANO2 in the dendrites could possibly counteract excitatory inputs from climbing fibers, a hypothesis that has to be tested in future studies when Purkinje cell-specific knockout models for ANO2 become available.

Even though ANO2 seems to affect the excitability of Purkinje cells only mildly, the importance of ANO2 for the olivo-cerebellar system has been shown by the observation that *Ano2*^*-/-*^ mice show gait abnormalities and perform poorly in cerebellum-dependent motor-coordination tasks, such as running-wheel and rotarod tests [11]. Moreover, *Ano2*^*-/-*^ mice have impaired motor-learning abilities, revealed by the eyeblink conditioning paradigm [9]. Eyeblink conditioning relies on a transient suppression of simple spike activity that seems to be induced by a combination of climbing fiber input and inhibition from molecular layer interneurons [49]. Recently, an increase in intrinsic excitability of Purkinje cells, observed in a mouse line lacking the calcium-gated potassium channel SK2 in Purkinje cells, has been associated with impaired eyeblink conditioning [50]. Moreover, the hyperpolarization after a burst of simple spikes is decreased after eyeblink conditioning [51]. ANO2 is among the factors controlling the eyeblink response, because several of the features contributing to proper eyeblink conditioning appear to be influenced by ANO2-chloride channels. Thus, the behavioral phenotypes of *Ano2*^*-/-*^ mice are in line with the notion that ANO2 channels contribute to motion control.

**Fig 9:**
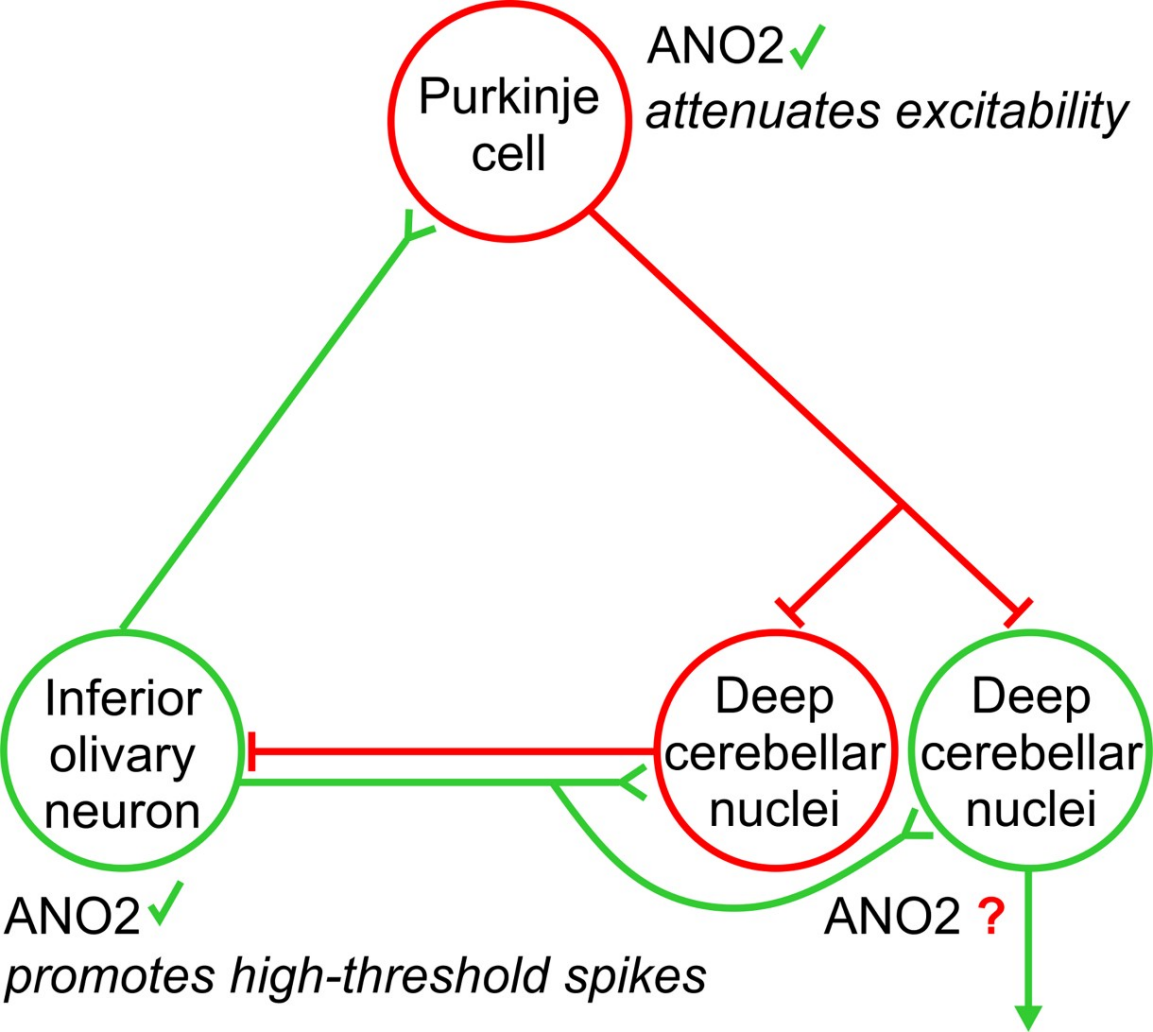
ANO2 in the olivo-cerebellar circuit. Schematic illustration of a cerebellar module summarizing the recent findings about the role of ANO2 in the olivocerebellar cortex. Glutamatergic neurons are depicted in green and GABAergic neurons in red. ANO2 has been shown to be expressed in inferior olivary neurons, where it accelerates repolarization after high-threshold calcium spikes and, thus, promotes the generation of these calcium spikes [9]. In Purkinje cells, ANO2 attenuates excitability upon strong activation. First indications of an expression of ANO2 in neurons of the deep cerebellar nuclei is presented in Fig 1A, but functional involvement in the cerebellar module awaits investigation.

The present study adds evidence to the emerging view that ANO2-mediated chloride currents subserve important functions in modulating neuronal excitability. The results of the present study, combined with the previous results on the inferior olivary nucleus [9], establish for the first time an involvement of ANO2-chloride channels in the modulation of excitability in two different neurons that are directly connected to each other within a neuronal network (Fig 9). Future studies may include the activity of neurons in the deep cerebellar nuclei of *Ano2*^*-/-*^ mice, which receive the output signal of Purkinje cells. Such studies may help to better understand this network effect of ANO2 and its implications for cerebellum-dependent motor coordination and learning tasks.

## Supporting information

S1 Fig Overview of expression of *Ano2* mRNA in neuronal tissue. Fold expression values obtained with qPCR studies of *Ano2* mRNA from different neuronal tissues normalized on the expression of brainstem tissue containing the inferior olivary nucleus. The two different tissue samples of the cerebellum are highlighted in green. IO = inferior olivary nucleus, OE = olfactory epithelium, Re = Retina, OB = olfactory bulb, CbH = cerebellar Hemisphere, CbV = cerebellar Vermis. Data are presented as mean ± 68%CI (IO: Fold expression 1.0, 68% CI 0.3–2.9; OE: Fold expression 10.1, 68% CI 4.0–30.0; Re: Fold expression 1.5, 68% CI 0.2–8.0; OB: Fold expression 0.1, 68% CI 0.07–0.18; CbH: Fold expression 0.3, 68% CI 0.1–1.4; CbV: Fold expression 0.5, 68% CI 0.1–1.7).
(TIF)

S2 Fig Spontaneous simple spike activity and basic electrical properties are unaltered by the elevation of E_Cl_. (A), (B) Spontaneous simple spike activity without (A) and with (B) 20 μM gabazine is unaffected by the elevation of E_Cl_ to the simple spike threshold (A: One-way ANOVA F(3, 54) = 1.2797, *p* = 0.2906; B: One-way ANOVA F(3, 90) = 1.7405, *p* = 0.1644). As expected, the spontaneous simple spike activity increases in the presence of gabazine (two-way ANOVA: Effect of gabazine: F(1, 144) = 11.34, *p* < 0.0001; recording condition x gabazine effect interaction: F(3, 144) = 0.73, *p* = 0.538) (C) The input resistance of the Purkinje cells, which was calculated using a test pulse in the voltage clamp configuration, is comparable between wildtype (black) and *Ano2*^*-/-*^ mice (red) and is unaltered by the elevation of E_Cl_ (Kruskal-Wallis test H(3) = 6.126, *p* = 0.106). (D) The threshold of the first simple spike is comparable between wildtype (black) and *Ano2*^*-/-*^ mice (red) for each of the depolarizing current injections and is unaltered by the elevation of E_Cl_ (one-way ANOVA; threshold activation: F(3, 104) = 0.6491, *p* = 0.5835; moderate activation: F(3, 125) = 0.4672, *p* = 0.7057; strong activation: F(3, 102) = 0.9072, *p* = 0.4404).
(TIF)

S3 Fig After hyperpolarization amplitude and time-to-minimum is comparable between wildtype and *Ano2*^*-/-*^ mice. Amplitude (left) and time-to-minimum (right) of hyperpolarization after threshold activation is comparable between wildtype (black) and *Ano2*^*-/-*^ mice (red) for both threshold (A) and moderate activation (B) of Purkinje cells (A; amplitude E_Cl_ -87 mV: wt -6.4 ± 2.1 mV, n = 37 cells of N = 8 animals, *Ano2*^*-/-*^ -6.1 ± 2.5 mV, n = 43 N = 11, Student’s *t*-test, *p* = 0.5860; amplitude E_Cl_ -48 mV: wt -5.7 mV IQR -7.2 - -4.9, n = 31 N = 6, *Ano2*^*-/-*^ -5.2 mV IQR -7.0 - -4.1, n = 36 N = 8, Wilcoxon-Mann-Whitney test, *p* = 0.661; time-to-minimum E_Cl_ -87 mV: wt 216.9 ± 61.3 ms, n = 37 N = 8, *Ano2*^*-/-*^ 222.0 ± 54.7 ms, n = 43 N = 11, Student’s *t*-test, *p* = 0.7012; time-to-minimum E_Cl_ -48 mV: wt 196.1 ± 49.9 ms, n = 31 N = 6, *Ano2*^*-/-*^ wt 218.3 ± 58.3 ms, n = 36 N = 8, Student’s *t*-test, *p* = 0.0988) (B; amplitude E_Cl_ -87 mV: wt -8.6 ± 3.0 mV, n = 34 N = 8, *Ano2*^*-/-*^ -7.8 ± 3.0 mV, n = 30 N = 9, Student’s *t*-test, *p* = 0.2837; amplitude E_Cl_ -48 mV: wt -8.2 ± 2.8 mV, n = 29 N = 6, *Ano2*^*-/-*^ -7.6 ± 2.2 mV, n = 30 N = 8, Student’s *t*-test, *p* = 0.3021; time-to-minimum E_Cl_ -87 mV: wt 200.8 ± 60.7 ms, n = 34 N = 8, *Ano2*^*-/-*^ 197.8 ± 43.4 ms, n = 30 N = 9, Student’s *t*-test, *p* = 0.8164; time-to-minimum E_Cl_ -48 mV: wt 176.4 ms IQR 135.0–236.7, n = 29 N = 6, *Ano2*^*-/-*^ wt 171.9 ms IQR 145.8–223.0, n = 30 N = 8, Wilcoxon-Mann-Whitney test, *p* = 0.735).
(TIF)

S4 Fig The paired-pulse ratio is comparable before and after depolarization in wildtype and *Ano2*^*-/-*^ mice. The paired-pulse ratios of eIPSC amplitudes of Purkinje cells before and after depolarization is comparable in both wildtype (black) and *Ano2*^*-/-*^ mice (red) (wildtype: Before depolarization 0.77 ± 0.18, after depolarization 0.96 ± 0.35, n = 9 cells of N = 6 animals, paired Student’s *t*-test *p* = 0.1269; *Ano2*^*-/-*^: Before depolarization 0.84 ± 0.26, after depolarization 0.77 ± 0.38, n = 14 cells of N = 9 animals, paired Student’s *t*-test *p* = 0.5584).
(TIF)

S1 File Raw data. (XLSX)
