# Supplementary figures and images for: Anoctamin 2-chloride channels reduce simple spike activity and mediate inhibition at elevated calcium concentration in cerebellar Purkinje cells
Source: PLoS One. 2021 Mar 2;16(3):e0247801. doi: 10.1371/journal.pone.0247801 (PMC7924762; doi:10.1371/journal.pone.0247801)

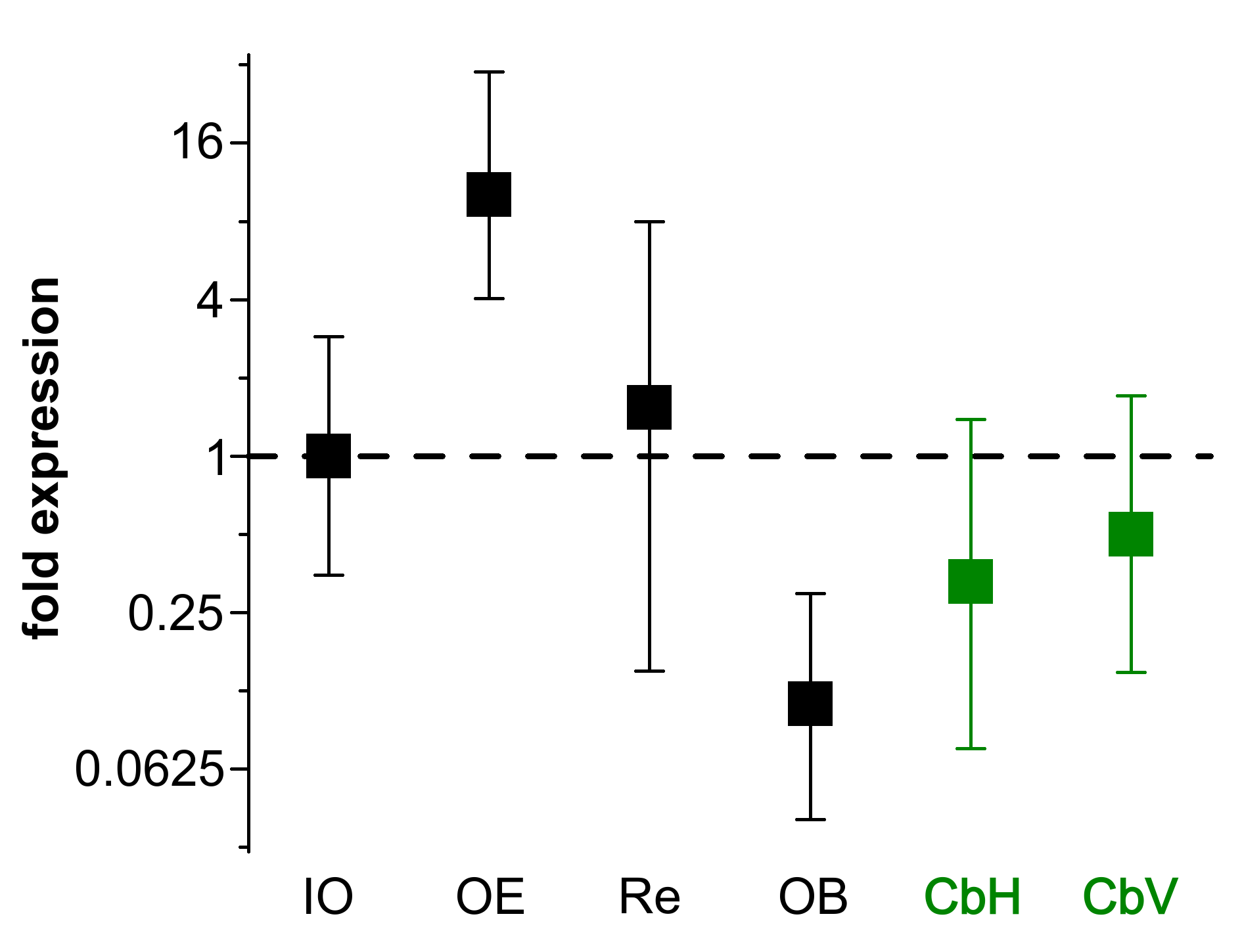

Supplement: S1 Fig — Fold expression values obtained with qPCR studies of Ano2 mRNA from different neuronal tissues normalized on the expression of brainstem tissue containing the inferior olivary nucleus. The two different tissue samples of the cerebellum are highlighted in green. IO = inferior olivary nucleus, OE = olfactory epithelium, Re = Retina, OB = olfactory bulb, CbH = cerebellar Hemisphere, CbV = cerebellar Vermis. Data are presented as mean ± 68%CI (IO: Fold expression 1.0, 68% CI 0.3–2.9; OE: Fold expression 10.1, 68% CI 4.0–30.0; Re: Fold expression 1.5, 68% CI 0.2–8.0; OB: Fold expression 0.1, 68% CI 0.07–0.18; CbH: Fold expression 0.3, 68% CI 0.1–1.4; CbV: Fold expression 0.5, 68% CI 0.1–1.7). (TIF) [file pone.0247801.s001.tif]

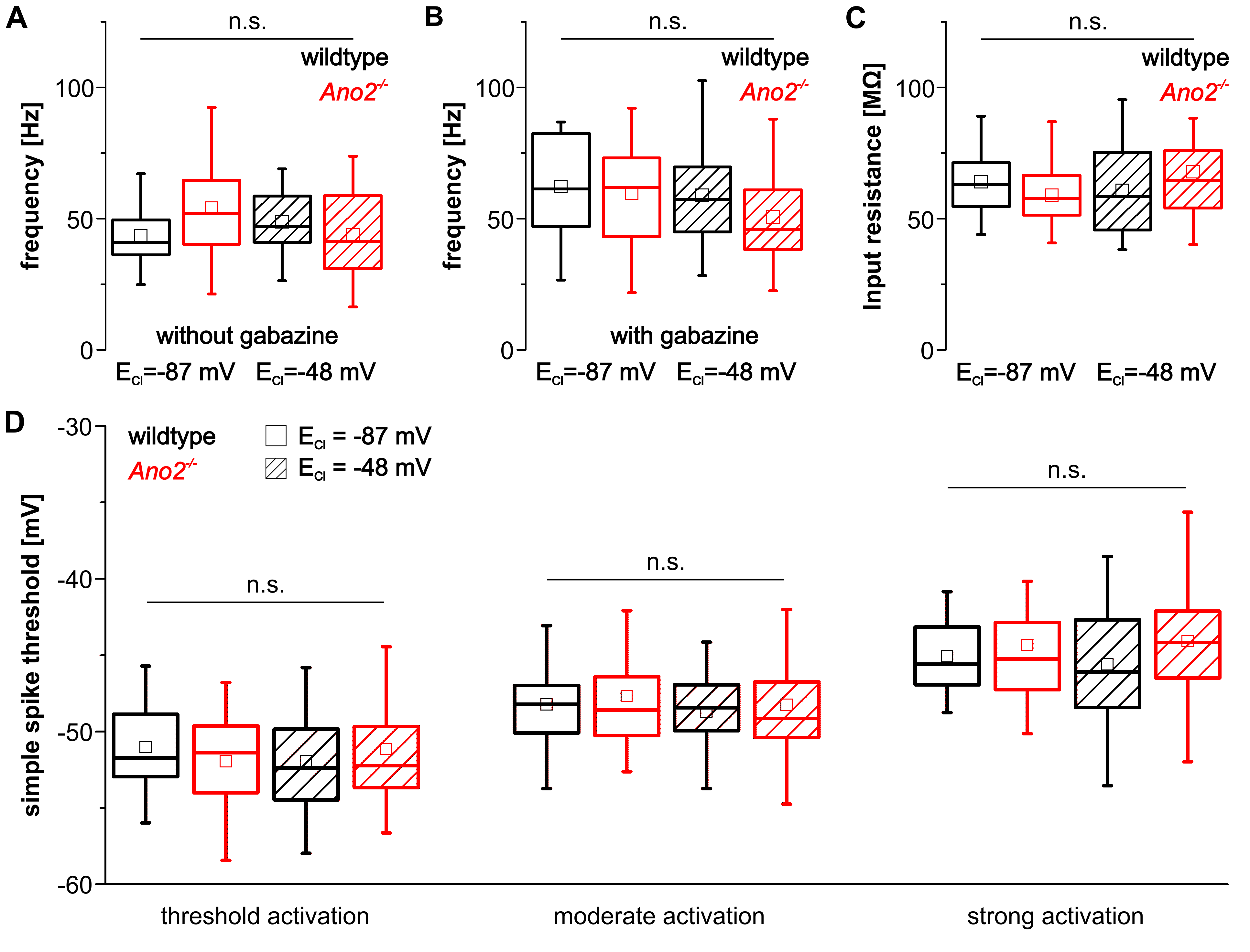

Supplement: S2 Fig — (A), (B) Spontaneous simple spike activity without (A) and with (B) 20 μM gabazine is unaffected by the elevation of ECl to the simple spike threshold (A: One-way ANOVA F(3, 54) = 1.2797, p = 0.2906; B: One-way ANOVA F(3, 90) = 1.7405, p = 0.1644). As expected, the spontaneous simple spike activity increases in the presence of gabazine (two-way ANOVA: Effect of gabazine: F(1, 144) = 11.34, p < 0.0001; recording condition x gabazine effect interaction: F(3, 144) = 0.73, p = 0.538) (C) The input resistance of the Purkinje cells, which was calculated using a test pulse in the voltage clamp configuration, is comparable between wildtype (black) and Ano2-/- mice (red) and is unaltered by the elevation of ECl (Kruskal-Wallis test H(3) = 6.126, p = 0.106). (D) The threshold of the first simple spike is comparable between wildtype (black) and Ano2-/- mice (red) for each of the depolarizing current injections and is unaltered by the elevation of ECl (one-way ANOVA; threshold activation: F(3, 104) = 0.6491, p = 0.5835; moderate activation: F(3, 125) = 0.4672, p = 0.7057; strong activation: F(3, 102) = 0.9072, p = 0.4404). (TIF) [file pone.0247801.s002.tif]

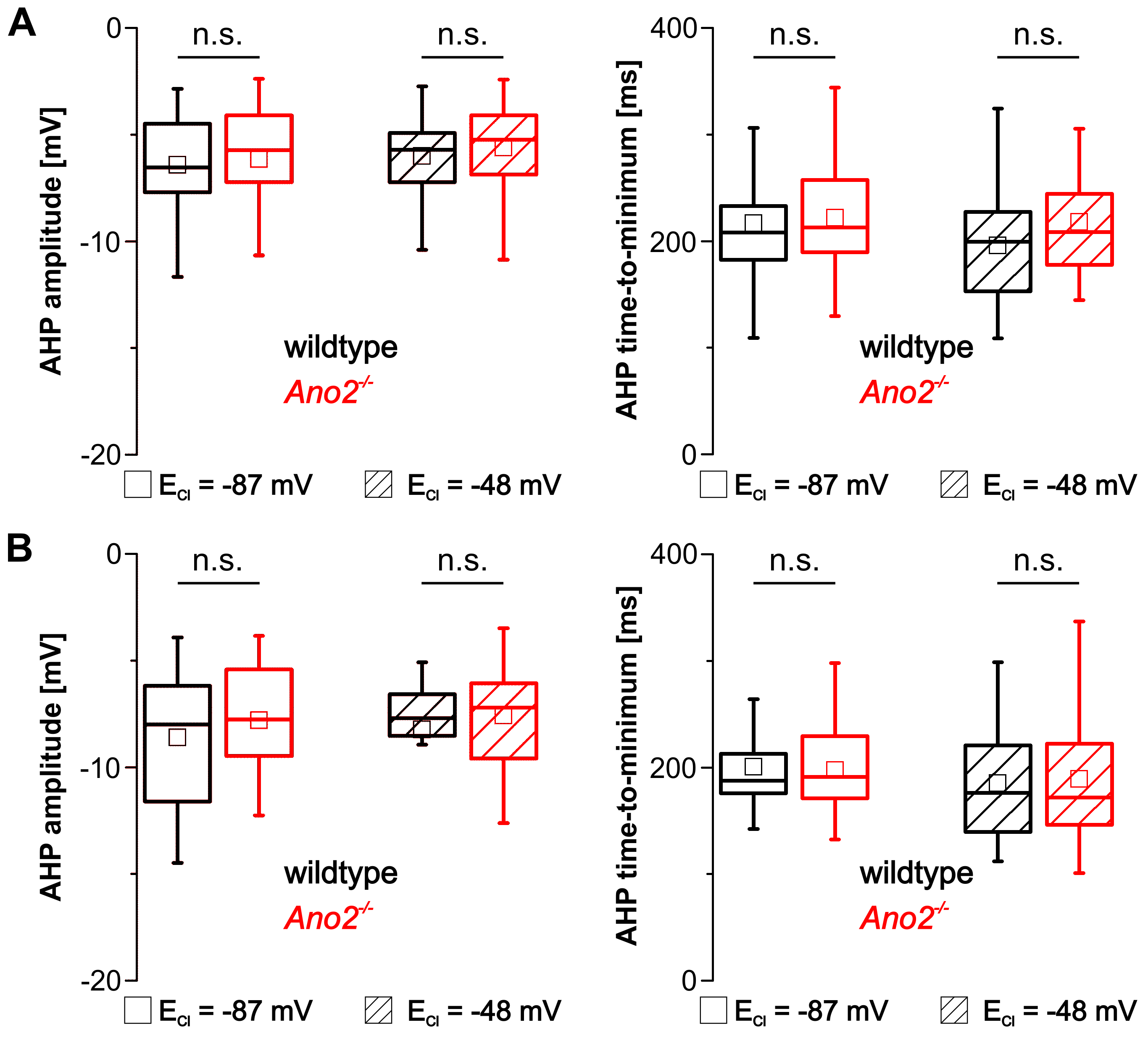

Supplement: S3 Fig — Amplitude (left) and time-to-minimum (right) of hyperpolarization after threshold activation is comparable between wildtype (black) and Ano2-/- mice (red) for both threshold (A) and moderate activation (B) of Purkinje cells (A; amplitude ECl -87 mV: wt -6.4 ± 2.1 mV, n = 37 cells of N = 8 animals, Ano2-/- -6.1 ± 2.5 mV, n = 43 N = 11, Student’s t-test, p = 0.5860; amplitude ECl -48 mV: wt -5.7 mV IQR -7.2 - -4.9, n = 31 N = 6, Ano2-/- -5.2 mV IQR -7.0 - -4.1, n = 36 N = 8, Wilcoxon-Mann-Whitney test, p = 0.661; time-to-minimum ECl -87 mV: wt 216.9 ± 61.3 ms, n = 37 N = 8, Ano2-/- 222.0 ± 54.7 ms, n = 43 N = 11, Student’s t-test, p = 0.7012; time-to-minimum ECl -48 mV: wt 196.1 ± 49.9 ms, n = 31 N = 6, Ano2-/- wt 218.3 ± 58.3 ms, n = 36 N = 8, Student’s t-test, p = 0.0988) (B; amplitude ECl -87 mV: wt -8.6 ± 3.0 mV, n = 34 N = 8, Ano2-/- -7.8 ± 3.0 mV, n = 30 N = 9, Student’s t-test, p = 0.2837; amplitude ECl -48 mV: wt -8.2 ± 2.8 mV, n = 29 N = 6, Ano2-/- -7.6 ± 2.2 mV, n = 30 N = 8, Student’s t-test, p = 0.3021; time-to-minimum ECl -87 mV: wt 200.8 ± 60.7 ms, n = 34 N = 8, Ano2-/- 197.8 ± 43.4 ms, n = 30 N = 9, Student’s t-test, p = 0.8164; time-to-minimum ECl -48 mV: wt 176.4 ms IQR 135.0–236.7, n = 29 N = 6, Ano2-/- wt 171.9 ms IQR 145.8–223.0, n = 30 N = 8, Wilcoxon-Mann-Whitney test, p = 0.735). (TIF) [file pone.0247801.s003.tif]

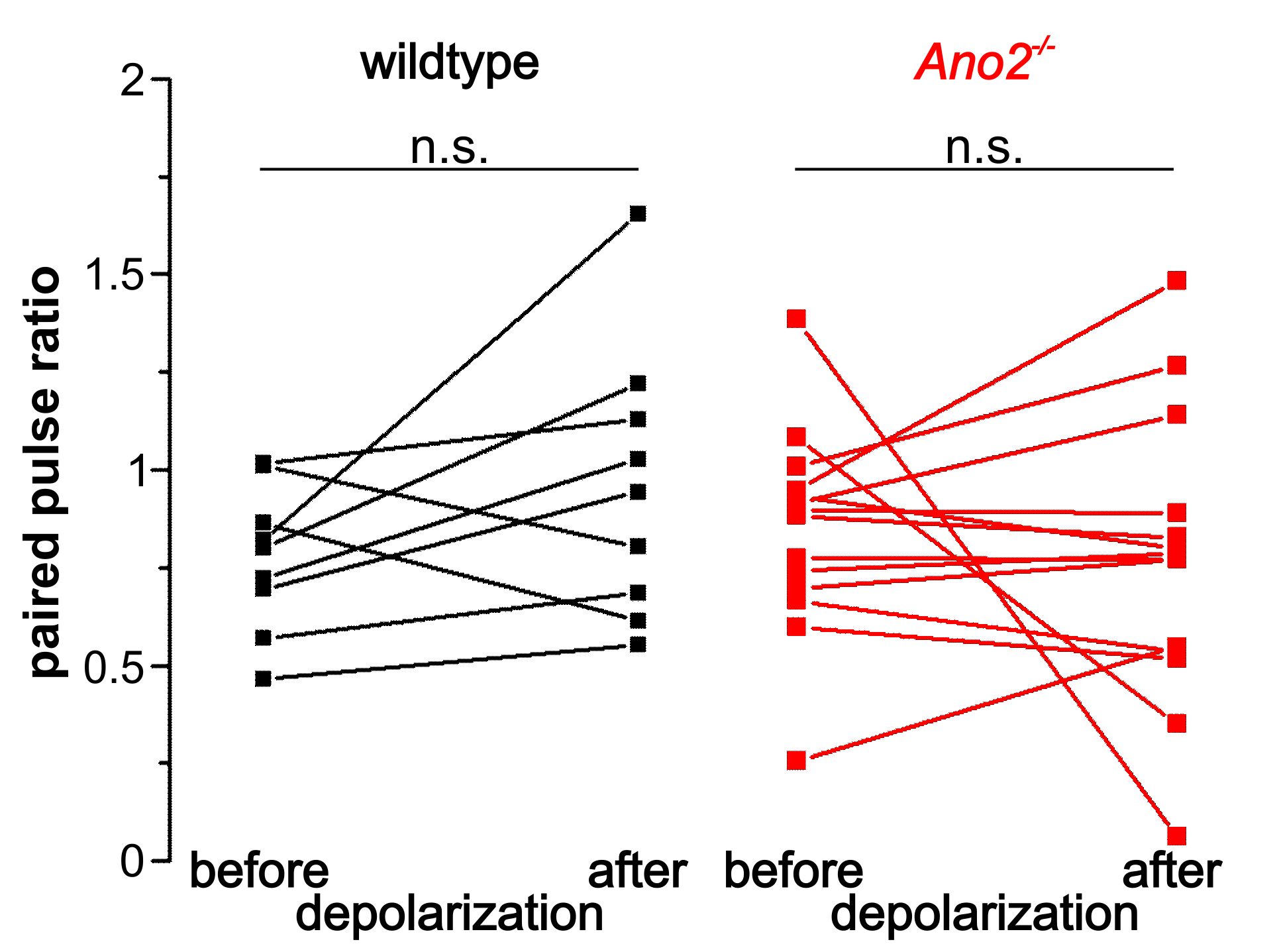

Supplement: S4 Fig — The paired-pulse ratios of eIPSC amplitudes of Purkinje cells before and after depolarization is comparable in both wildtype (black) and Ano2-/- mice (red) (wildtype: Before depolarization 0.77 ± 0.18, after depolarization 0.96 ± 0.35, n = 9 cells of N = 6 animals, paired Student’s t-test p = 0.1269; Ano2-/-: Before depolarization 0.84 ± 0.26, after depolarization 0.77 ± 0.38, n = 14 cells of N = 9 animals, paired Student’s t-test p = 0.5584). (TIF) [file pone.0247801.s004.tif]
